# Looking for trees in the forest: summary tree from posterior samples

**DOI:** 10.1186/1471-2148-13-221

**Published:** 2013-10-04

**Authors:** Joseph Heled, Remco R Bouckaert

**Affiliations:** 1 Department of Computer Science, University of Auckland, Auckland New Zealand; 2 Computational Evolution Group, University of Auckland, Auckland New Zealand

## Abstract

**Background:**

Bayesian phylogenetic analysis generates a set of trees which are often condensed into a single tree representing the whole set. Many methods exist for selecting a representative topology for a set of unrooted trees, few exist for assigning branch lengths to a fixed topology, and even fewer for simultaneously setting the topology and branch lengths. However, there is very little research into locating a good representative for a set of rooted time trees like the ones obtained from a BEAST analysis.

**Results:**

We empirically compare new and known methods for generating a summary tree. Some new methods are motivated by mathematical constructions such as tree metrics, while the rest employ tree concepts which work well in practice. These use more of the posterior than existing methods, which discard information not directly mapped to the chosen topology. Using results from a large number of simulations we assess the quality of a summary tree, measuring (a) how well it explains the sequence data under the model and (b) how close it is to the “truth”, i.e to the tree used to generate the sequences.

**Conclusions:**

Our simulations indicate that no single method is “best”. Methods producing good divergence time estimates have poor branch lengths and lower model fit, and vice versa. Using the results presented here, a user can choose the appropriate method based on the purpose of the summary tree.

## Background

Bayesian Markov Chain Monte Carlo (MCMC) analysis provides powerful and popular techniques for performing phylogenetic analysis. The result of such an analysis is a set of trees drawn from the posterior distribution. The set of correlated draws is often condensed into a single tree for visualistion, comprehension, annotations and presentation. When most trees agree in topology and branch lengths, the most frequent tree topology, properly annotated, can give a fair representation of the posterior distribution.

However, a single tree can be misleading, especially when the agreement between posterior trees is small. Posterior tree topologies can be reduced to a set of common sub-topologies [1], but this is also a fragmented view of the posterior. Tree drawing programs such as FigTree [2] can annotate internal nodes with the clade posterior support (the fraction of posterior trees containing the clade), and the credible interval of internal node ages.

Still, the choice of any specific topology highlights one alternative at the expense of others. The tree drawing program DensiTree draws all posterior trees transparently [3]. Where most trees agree in topology and node height, lines are close to each other and distinct edges appear, while areas of uncertainty in topology or heights remain a blur. The composite image allows a direct assessment of posterior support and node height uncertainty by visual inspection. But even the display of the full posterior can be hard to interpret when the uncertainty gets large, and a summary tree overlaid on top can be useful in such situations.

There are many ways of obtaining a “representative” tree topology from a collection of trees. One group of methods look for consensus among the trees using splits, clusters or rooted triplets/quartets present in posterior trees [4]. The TreeAnnotator utility in BEAST [5] uses the clade frequencies as estimated by the posterior to score each tree, selecting the rooted topology of the highest scoring tree amongst the set. This results in a fully resolved topology with a non zero support, in contrast to consensus methods which often produce unresolved trees. A recently published method uses conditional clade splits probabilities to compute a probability for each posterior tree [6].

However, selecting a representative topology is only the first step in generating a summary tree from the output of programs such as BEAST. BEAST trees are rooted with branches proportional to time, as is the summary tree. In the second step, TreeAnnotator assigns a divergence time for each clade using the ages of matching clades from the posterior. If the number of trees containing the clade is small, the divergence time estimate can have high variance which may result in negative branch lengths. Clades in trees from the MCMC samples which do not appear in the summary tree are essentially ignored, and sometimes a large proportion of the posterior goes unrepresented. Ignoring non-matching parts appears to be the accepted practice and is used in the SumTrees utility in DendroPy [7].

In this paper we describe several new ways for building rooted summary trees. These new constructions use more of the information contained in the posterior even when the disagreement between posterior trees is high. Some of the methods are based on rooted tree distances, and are similar in spirit to the method developed by Huggins et al. for unrooted trees [8]. We perform an extensive simulation study and compare the trees from all methods using multiple criteria. Summary trees are assessed with respect to the posterior and by their distance to the tree used in generating the sequence data. The methods are implemented in biopy [9], and integrated with DensiTree, making it easy to examine the summary tree in the context of the full posterior.

## Methods

### Definitions and notations

We define a rooted tree as a collection of clades with ages. Specifically, a tree is a strict hierarchy of clades, where each clade is a subset of the taxa, and a non-negative age is associated with each clade.

Formally, a tree *T* is a triplet (L,ℂ,h), where $L=\left\{x_{1},x_{2},\ldots ,x_{l}\right\}$ is the set of taxa and $\mathbb{C}=\left\{C_{1},C_{2},\ldots ,C_{n}\right\}$ is a set of clades. Each clade *C*_*i*_⊆*L* is a subset of taxa, and $h:\mathbb{C}\to {\mathbb{R}}_{\geq 0}$ is a function assigning an age to the clade. The set ℂ describes only the clades hierarchy and is referred to as the tree topology. Sometimes we shall use *c*∈*T* as a shorthand for T=(L,ℂ,h)andc∈ℂ (“clade *c* is present in tree *T*”).

To qualify as a tree, the following conditions must hold: 

i The tree contains all leaves: $\forall_{i}\{x_{i}\}\in \mathbb{C}$.

ii The tree contains a root: L∈ℂ.

iii Strict hierarchy of clades: for any two clades $c_{1},c_{2}\in \mathbb{C}$, either *C*_1_⊂*C*_2_, *C*_2_⊂*C*_1_ or *C*_1_∩*C*_2_=*∅*. (Note that *C*_1_⊂*C*_2_ implies *C*_1_≠*C*_2_, otherwise we write *C*_1_⊆*C*_2_.)

iv Non-Negative branches: for $c_{1},c_{2}\in \mathbb{C}$, *c*_1_⊂*c*_2_⇒*h*(*c*_1_)≤*h*(*c*_2_).

For any clade *c*, the elements in the set A(c)={y∈ℂ:c⊂y} are called ancestors of *c*, and the minimal element *P*(*c*) in *A* is the parent of *c*. Every clade except the root has a parent and by association a branch to its parent with length *b*(*c*)=*h*(*P*(*c*))−*h*(*c*). For convenience, the branch length of a subset not in  is defined as zero. Any subset of taxa *x* has a *Most Recent Common Ancestor* in the tree, the minimal clade containing all members of *x*. Formally, ca(*x*) is the minimal element of {y∈ℂ:x⊆y}. For brevity we omit the tree when the context is clear, and use ca(*c*,*T*) to explicitly associate the clade with the tree *T*.

Extending the domain of *b*(·) to all taxa subsets simplifies definitions involving sets of trees with different topologies. We extend *h*(·) for the same reason and define the age of any subset *x*⊆*L* to be the age of the common ancestor of *x*,$\bar{h}(x)=h(\text{ca}(x))$.

Using $\bar{h}$, we define the *heights error*, a discrepancy score between clade ages of T=(L,ℂ,h) and a reference tree *T*_*ref*_,

(1) $$\varepsilon_{H}(T,T_{\text{ref}})=\underset{c\in \mathbb{C}}{\sum }|h(c)-{\bar{h}}^{\text{ref}}(c)|.$$

The heights error is the total sum of clade age errors, whether they appear in the reference tree or not. The age of a clade which is not in the reference tree is taken to be the age of the MRCA of the clade taxa, which spans a larger clade in the reference tree. Note that the definition is not symmetric. Alternatively we define the *divergence times error* which focuses on the time lineages split from each other. The divergence time for any clade *x*⊆*L* is the mean divergence time of all pairs of *x*. Formally, we start with the pairs of taxa which split at the clade; those are the pairs in *x* whose common ancestor is the clade,

(2) $$D(x,T)=\left\{\left\{a,b\right\}\in x:\text{ca}(T,\left\{a,b\right\})=x\right\}.$$

Now the average split time is the mean of all pair splits,

(3) $$\tilde{h}(x,T)=|D(x,T){|}^{-1}\underset{a,b\in D(x,T)}{\sum }\bar{h}(\{a,b\}).$$

Finally, The total error is,

(4) $$\varepsilon_{D}(T,T_{\text{ref}})=\underset{c\in T}{\sum }|h(c)-\tilde{h}(c,T_{\text{ref}})|$$

The clade and divergence errors are equal for trees with the same topology, but they differ when topologies disagree, and the difference usually increases with the distance in topology.

The *clades missed error* counts the number of clades in *T*_*ref*_ not present in *T*,

(5) $$\varepsilon_{\text{cm}}(T,T_{\text{ref}})=|{\mathbb{C}}^{\text{ref}}|-|\left\{c\in \mathbb{C}\cap {\mathbb{C}}^{\text{ref}}:b(c)>0\right\}|.$$

This number is equal to (half) the Robinson-Foulds tree distance [10] when *T* has no zero length branches. A clade with a zero branch does not count as a match because it is potentially confused with its parent. The *clades called error* scores a 1 for correctly called clades and a -1 penalty for incorrectly called clades,

(6) $$\begin{array}{l} \varepsilon_{\text{cc}}(T,T_{\text{ref}})=|\left\{c\in \mathbb{C}\setminus {\mathbb{C}}^{\text{ref}}:b(c)>0\right\}|\\ \;-|\left\{c\in \mathbb{C}\cap {\mathbb{C}}^{\text{ref}}:b(c)>0\right\}|. \end{array}$$

A *tree set*$T=\left\{T_{1},T_{2},\ldots ,T_{k}\right\}$ is a set of trees on shared taxa. Typically those sets are samples from a Bayesian analysis, and we define the posterior frequency *F*(*x*) of *x*⊆*L* as the fraction of times *x* is present as a clade in the trees:

(7) $$F(x)=\frac{1}{\left|T\right|}|\left\{T\in T:x\in T\right\}|.$$

The posterior frequency of a subset not in any of the trees is zero.

### Distance between trees

The Rooted Branch Score (RBS) measures the distance between two rooted time trees, and is the total sum of the difference in branch lengths of matching clades. This definition is motivated by the distance between unrooted trees [11], but the space of rooted trees is more complex than its unrooted counterpart since branch lengths are not free to vary independently of each other [12]. Since by convention the branch length of a missing clade is zero, any clade present only in one tree contributes its total length to the score.

Formally, for $T_{1}=(L,{\mathbb{C}}^{1},h^{1})$ and $T_{2}=(L,{\mathbb{C}}^{2},h^{2})$ we have,

(8) $$\text{RBS}(T_{1},T_{2})=\underset{c\in {\mathbb{C}}^{1}\cup {\mathbb{C}}^{2}}{\sum }|b^{\left(1\right)}(c)-b^{\left(2\right)}(c)|.$$

The Squared Branch Score (SRBS) is similar, but taking the square of the difference instead of the absolute value,

(9) $$\text{SRBS}(T_{1},T_{2})=\underset{c\in {\mathbb{C}}^{1}\cup {\mathbb{C}}^{2}}{\sum }{\left(b^{\left(1\right)}(c)-b^{\left(2\right)}(c)\right)}^{2}.$$

The Heights Score (HS) takes the difference between clade ages instead of branches. Like the RBS, branches appearing in only one tree are added to the sum,

(10) $$\begin{array}{l} \text{HS}(T_{1},T_{2})=\underset{c\in {\mathbb{C}}^{1}\cap {\mathbb{C}}^{2}}{\sum }|h^{\left(1\right)}(c)-h^{\left(2\right)}(c)|+\\ \;\underset{c\in {\mathbb{C}}^{1}\setminus {\mathbb{C}}^{2}}{\sum }b(c)+\underset{c\in {\mathbb{C}}^{2}\setminus {\mathbb{C}}^{1}}{\sum }b(c). \end{array}$$

The heights score is a (non-optimal) edit distance, where the score is the total sum of a sequence of moves which transform one tree into the other. Each move involves sliding an internal node, and two nodes may “merge” into one when they meet.

The Rooted Agreement Score (RAS) measures the disagreement between branches by treating them as intervals. Two branches may be of the same length and still contribute to the distance if they span different intervals as measured from the time of the tips. The score, when divided by the sum of the length of the two trees, is the probability that a random point chosen uniformly on one of the trees has a corresponding point on the other tree. Formally,

(11) $$\begin{array}{l} \text{RAS}(T_{1},T_{2})=\underset{c\in {\mathbb{C}}^{1}\cap {\mathbb{C}}^{2}}{\sum }\mu \left({\vec{b}}^{\left(1\right)}(c)△{\vec{b}}^{\left(2\right)}(c)\right)\\ \;+\underset{c\in {\mathbb{C}}^{1}\setminus {\mathbb{C}}^{2}}{\sum }b(c)+\underset{c\in {\mathbb{C}}^{2}\setminus {\mathbb{C}}^{1}}{\sum }b(c), \end{array}$$

where $\vec{b}(x)$ is the interval spanned by the clade branch, $\vec{b}(x)=[h(x),h(x)+b(x)]$ and △ is the symmetric difference operator, that is

(12) $$\begin{array}{l} \;\mu ([\;l_{1},h_{1}]△[\;l_{2},h_{2}])\;=\;(h_{1}-l_{1})+(h_{2}-l_{2})\\ \;-\;2\max(\min(h_{1},h_{2})\;-\;\max(l_{1},l_{2}),0). \end{array}$$

RBS and RAS are metrics in tree space, while SRBS and HS are not. RBS is a metric since branches can be mapped to the vector space ${\mathbb{R}}^{2^{n}-1}$[8], and a similar argument works for RAS. However, we only require that distances are semimetrics and make no use of the triangle inequality.

### Summary trees

#### BEAST Tree annotator

The *Tree Annotator* utility in BEAST generates a summary tree using a two stage procedure. First, each posterior tree is assigned a score based on topology. The *Clade Credibility* of a tree is the product of posterior frequencies (equation (7)) of all clades in the tree,

$$\mathrm{CC}(T=(L,C,h))=\underset{c\in C}{\prod }F(c).$$

The *Maximal Clade Credibility* (MCC) tree is the tree with the highest score, and we shall refer to its topology as the MCC topology. In the second step, each clade is assigned an age based on the clade age in posterior trees. Formally, the age is set as either the mean or the median of the set of ages

$$H(c,T)=\left\{h_{i}(c):(L,{\mathbb{C}}_{i},h_{i})\in T\;\text{and}\;c\in {\mathbb{C}}_{i}\right\}.$$

Since each age is set independently, the end result is not guaranteed to be a tree (condition iii). A few “negative branches” are not an unusual occurrence in trees with a medium to large number of taxa and moderate posterior uncertainty.

#### Minimum distance trees

The distance between the tree set  and the tree *T* is defined as the mean distance of *T* to all members of ,

(13) $$d(T,T)=|T{|}^{-1}\underset{t_{i}\in T}{\sum }d_{T}(T,t_{i}),$$

where *d*_*T*_ is one of the tree scores defined previously. A *Minimum Distance Tree* is a tree which minimizes d(T,T). While the definition is simple and natural, the details are not. First, the minimal tree is not necessarily unique; there might be several or even an infinite number of minimal trees in some cases. Second, with anything more than a few taxa the space of trees is vast and topologically complex, so there is no guarantee of finding the minimal tree. We therefore limit the search to the topologies present in the posterior, and designate this approach by a lowercase ‘m’ followed by the distance method (mRBS, mRAS, etc). However, even this can be time consuming when the posterior contains many topologies, and in addition we examine a family of methods which consider just a single topology, using one of the heuristics outlined in the next section. The details about the algorithm for searching the best branch assignment for a specific topology are in Appendix 2.

#### Selecting a topology

All of the two stage methods we considered selects a topology first and assign branch lengths conditional on that topology. We examined three alternatives to the MCC for selecting a topology.

The first alternative uses the recently published Conditional Clade Probability Distribution (CCD). The CCD computes a probability for each tree based upon the posterior probability of the splits in the tree, conditional on the clade posterior frequency [6]. The second is a the Total Clade Branch (TCB), which assigns a score to each clade in the tree equal to the total length of matching branches in the posterior. The total length reflects the support for a clade by combining both the frequency (the number of trees with the clade) and confidence, under the assumption that longer branches are more likely to be “real” than shorter branches. The third is the Highest Posterior Frequency (HPF), which picks the topology of the tree most frequent in the posterior. To break ties, the HPF picks the tree whose height is closest to the mean root height of the posterior.

#### CA Tree

Negative branches in the TreeAnnotator tree result from using a different subset of posterior trees for estimating each clade age. In the *Common Ancestor Tree* (CAT), every clade x∈ℂ is assigned an age using the mean of the clade age in **all** posterior trees. Formally,

(14) $$h(c):=|T{|}^{-1}\underset{T_{i}\in T}{\sum }\bar{h}(c,T_{i})$$

The generated ages always produce a tree, since $x\subset y\Rightarrow \bar{h}(x,T_{i})\leq \bar{h}(y,T_{i})$. Unlike TreeAnnotator, which may end up using a small number of values for some clades, CAT uses |T| posterior values for estimating the age of each clade.

#### Taxa partitions tree

We now present the Taxa Partition (TP) tree, a single stage method which does not commit to a particular topology before assigning ages. The TP is inspired by the tree operator described by Mau *et al*[13]. In this representation each internal node is assigned a left/right orientation, inducing a linear order on the taxa and positioning each internal node between two tips (Figures one and two in [13]). We reverse the process by first ordering the taxa, then using the posterior to assign the ages between tips and finally reconstructing the tree topology from the ages.

For a given ordering of taxa, each posterior tree provides ages according to its topology. A clade contributes an age if it spans an unbroken range in the ordering. For example, for the order [a b c d], the tree (((a,b),c),d) contributes the age of (a,b) to [a | bcd], the age of ((a,b),c) to [ab | cd] and the root height to [abc | d]. The tree ((a,d),(b,c)) contributes only the age of (b,c) to [ab | cd]. (a,((d,b),c)) contribute only its root height to [a | bcd].

After collecting ages for all splits from the posterior, a point estimate of the height at each split is used to build the tree. The precise definitions are given in Appendix 2.

TP incorporates clade ages from competing topologies before committing to the final topology. For example, take the set with a mixture of two topologies, ((a,b),c) and (a,(b,c)). With the obvious ordering [a b c], TP uses all ages in every tree, and the choice between the two topologies is determined by the age of the [ab | c] and [a | bc] splits. If [ab | c] is higher we end up with ((a,b),c), otherwise with (a,(b,c)).

Finding an optimal ordering is hard. Assigning an orientation which minimizes the distance between taxa orders of just two trees is NP complete [14]. We use a fast heuristic which proved effective in practice: build a distance matrix for pairs of taxa and use simple clustering to build the ordering. The distance between taxa *a* and *b* in each tree is the size of the clade of their common ancestor, *d*(*a*,*b*)=|ca({*a*,*b*})|. The overall distance is the mean of pair distances over all posterior trees. The clustering starts with each taxon in its own group, then progressively joins the two closest groups.

### Test cases

To evaluate the various methods we generated 2000 test cases, divided into 20 groups of 100 repeats. For each case, a tree with *n* tips was drawn from the Kingman coalescent distribution [15] with population size *N*_*e*_. All repeats shared the same *n* and *N*_*e*_, and each group was assigned one pair from the 5x4 grid formed by *n*=8,16,32,64,128 and *N*_*e*_=1,2,4,8.

A sequence of length 800bp was generated for the tips of the tree, starting with an ancestral sequence at the root and mutating the sequence along the branches using the Jukes-Cantor substitution model [16] with a mutation rate of 0.005. The sequences were analyzed using BEAST-2 [17] under the same model (Jukes-Cantor and a coalescent prior with constant population size). The tree and population size were estimated but the mutation rate was fixed at its true value. The chain was 2.2M steps, sampled every 2k steps. 200k of the initial samples were discarded (burn-in), leaving 1000 posterior samples. Those were used as input for building a summary tree by each of the methods under consideration.

The test trees contain 8 to 128 tips and range (on average) from a height of 0.01 substitutions to 0.08, or 2 to 16 million years for a nuclear mammalian gene. Sampling the posterior of such trees normally requires a longer MCMC chain, but here a relatively short one is sufficient. The data was generated under a simple model and the exact same model is used for inference, resulting in excellent mixing. Not only was the effective sample size high for all parameters, we made sure the clades were adequately sampled by running a second independent chain, starting with a different seed. We then computed the maximum of the absolute difference between posterior frequency of all clades; this number was well below 5% in most settings, and around 6% for the most diffuse case (128 tips and height of 0.01 substitutions).

The posterior for trees with 32 and more taxa was completely diffuse, with a distinct topology for each sample. Even the easiest cases (*n*=8 and *N*_*e*_=8) contained between 1 and 45 distinct topologies, with a mean of 6. Also note that even when the posterior has a single topology, a method may do better that others by setting more accurate branch lengths.

Summary trees were compared using two main criteria: accuracy in estimating ages and model fit. The first criteria was broken into 3 related error measures: accuracy in estimating the root height, accuracy in estimating clade ages (equation 1) and accuracy in estimating divergence times (equation 4). The second criteria was also divided into three: the log-likelihood of the sequence data given the tree (tree likelihood), the log-likelihood of the tree under the coalescent (coalescent likelihood), and the overall model fit, which is the sum of the tree and coalescent likelihood.

### How methods are ranked

The methods were compared by aggregating the results from all test cases. Let us take the root height as an example. For each test case, an error value is computed for each method by taking the absolute difference between the summery and true tree heights. Next, the methods are ranked by error using dense ranking (the 1-2-2-3 rule). Finally, the mean rank of each method is computed by averaging its rank over all 2000 tests.

This scoring procedure was repeated (bootstrapped) 4000 times. In each repeat 2000 test cases are sampled (with replacement) from the pool of 2000 test cases, and a mean score computed for each method. Method A was deemed better than B only if A’s mean ranking was greater than B’s in 90% (3600) of the bootstraps. The method gets a final score of 0 (best) if no other method is better, and a score of *R*+1 if there is a better method of score *R*.

The same process is repeated, using not the rankings of errors but the normalized error values. The normalization takes the errors of each case and transforms them to have a mean of 0 and a variance of 1. This ranks the methods by the magnitude of the error they make compared to other methods.

This may seem overly complex but making a fair comparison requires extra care. The methods and error measures are correlated in both obvious and subtle ways. Multiple criteria allows for a more nuanced comparison. Ideally, the particular mix of methods should not matter: adding a duplicate (or a very close variant) of one method should not penalize the ranking of lesser methods. Using dense ranking should minimize those effects. Strong correlations exist between the test settings (*N*_*e*_ and *n*) and the magnitude of errors, so aggregating results from the 20 groups requires some care. Rankings based on comparison alone are insensitive to those correlations, and the normalization of errors makes aggregation possible without going through the complex exercise of modeling the relations between settings. Another reason for using two rankings is that method A may be slightly better than B in (say) 60% of the cases, yet its errors in the other 40% are large. The difference between the two ranks would alert us to such situations.

Finally, any number of test cases, 2000 included, is small when considering the size of tree space. Bootstrapping provides some confidence that the results are stable and not due to random noise.

## Results

Table 1 lists the rankings of 22 methods for building summary trees. The table lists the comparison and error magnitude ranks for each of the 7 error measures: root height, clades missed and called (equations 5 and 6), ages and divergence times errors (equation 1 and 4), model fit, tree likelihood and coalescent likelihood. See Additional files 1 and 2 for the complete table and detailed per method rank graphs. Table 2 provides condensed rankings for the 22 methods together with performance statistics for each method obtained by averaging over the 2000 summary trees produced by each method.

**Table 1:** Rankings of methods for building a summary tree

**Method**	**RH**	**CME**	**CCE**	**CAE**	**DVE**	**MF**	**TLL**	**CLL**
TP(med)	1/3	0/0	12/9	8/8	7/5	3/3	0/0	3/3
TP(avg)	0/4	0/0	13/9	6/7	0/3	11/10	1/6	14/15
MED,TCB	1/0	3/3	10/7	6/4	6/6	9/7	8/11	9/9
MED,MCC	1/0	6/6	12/10	7/4	7/7	7/6	7/10	7/7
RBS,TCB	6/8	10/10	4/3	12/12	11/10	2/2	4/3	1/1
RBS,MCC	7/9	12/12	5/4	12/12	11/11	1/1	3/2	0/0
HSO,TCB	1/1	2/2	11/8	6/4	6/6	10/7	8/11	10/10
HSO,MCC	1/2	5/5	13/11	6/4	7/7	8/6	7/10	8/8
SRBS,TCB	3/5	8/7	8/5	6/5	1/2	12/9	6/9	13/13
SRBS,MCC	4/6	9/9	9/6	6/5	3/4	11/9	5/9	11/12
RAS,MCC	5/6	14/14	7/4	9/9	9/8	4/4	3/4	6/6
RAS,TCB	5/6	13/13	6/3	10/10	10/9	5/4	5/5	5/5
mSRBS	3/5	9/8	9/6	7/6	2/3	11/8	4/8	12/11
mRAS	5/7	15/15	7/4	11/11	10/9	6/5	6/7	4/4
mRBS	6/8	11/11	3/3	13/13	11/10	0/0	2/1	2/2
mHS	1/0	18/19	1/1	2/0	1/0	16/12	12/14	18/18
AVG,MCC	0/4	7/7	11/9	5/5	5/8	13/11	9/12	15/14
CAT,TCB	0/5	1/1	14/10	0/0	1/2	18/16	14/15	20/21
CAT,MCC	0/4	4/4	15/12	1/1	1/2	17/15	13/14	19/20
HS,TCB	2/2	17/17	2/2	4/3	4/1	15/14	11/17	16/16
HS,MCC	1/1	19/18	2/2	3/2	3/0	14/13	10/16	17/17
CONS(med)	1/0	16/16	0/0	3/2	8/8	17/14	15/13	19/19

Rankings of methods for building a summary tree from posterior samples. Both the comparison and error magnitude ranking are given for each method and 7 error measures (as a comparison/magnitude pair). The error measures are root height error (RH), clades missed (CME), clades called (CCE), clade ages errors (CAE), divergence times errors (DVE), model fit (MF) and tree likelihood/coalescent likelihood (TLL/CLL). Method names are as defined in the methods section, except for CONS,MED,AVG and HSO. CONS is the strict consensus tree with ages set by median estimates, as implemented by DendroPy. MED and AVG respectively use the median and average of clades ages from all matching trees in the posterior. HSO also uses the same clade ages, but uses the search algorithm utilized by the tree distance methods to find heights which minimize the total squared error.

**Table 2:** **Condensed rankings for methods in Table**1**with additional performance numbers**

**Method**	**TIMES**	**CME**	**CCE**	**MODEL**	**POLY**	**MF%**	**CAE%**	**CME%**
CAT,TCB	1	1	14	19	0.0%	45.2%	3.79%	36.33%
CAT,MCC	2	4	15	18	0.0%	45.3%	3.79%	36.46%
TP (avg)	5	0	13	11	0.0%	93.2%	4.37%	36.22%
TP (med)	12	0	12	1	0.0%	98.6%	4.50%	36.21%
								
SRBS,TCB	6	8	7	12	4.1%	91.8%	4.38%	36.64%
MED,TCB	7	3	9	8	1.1%	94.0%	4.36%	36.36%
HSO,TCB	8	2	10	10	1.1%	94.0%	4.36%	36.35%
MED,MCC	9	6	13	6	0.9%	94.6%	4.37%	36.48%
mSRBS	9	9	8	9	3.8%	92.1%	4.39%	36.80%
HSO,MCC	10	5	14	7	0.9%	94.6%	4.37%	36.48%
AVG,MCC	10	7	11	13	1.1%	84.2%	4.30%	36.49%
SRBS,MCC	11	10	8	11	4.2%	91.9%	4.39%	36.8%
								
mHS	0	19	1	16	29.8%	50.0%	3.97%	44.48%
HS,MCC	3	19	2	14	34.3%	54.9%	4.16%	44.27%
HS,TCB	4	18	2	15	34.5%	54.6%	4.18%	44.09%
CONS (med)	6	17	0	17	27.5%	46.3%	3.98%	43.00%
RAS,MCC	13	15	6	3	24.4%	93.1%	4.66%	42.45%
RAS,TCB	14	14	5	4	24.2%	92.7%	4.67%	42.26%
mRAS	15	16	6	5	24.5%	88.2%	4.74%	42.73%
RBS,TCB	16	11	4	2	23.4%	99.0%	4.82%	40.60%
mRBS	17	12	3	0	23.8%	99.1%	4.84%	40.80%
RBS,MCC	18	13	5	0	23.7%	99.0%	4.81%	40.94%

The ranks for RH, CAE and DVE were added to make the TIMES rank indicating fit of clade heights, and MF, TLL and CLL ranks added to make MODEL rank indicating fit of topology. The POLY column shows the mean number of branches with length zero, which effectively create a polytomy in the tree. The number of zero length branches in each tree were divided by the total number of branches to turn them in percentages so that they can be averaged over all 2000 test cases. The MF% column shows the mean percentile of the summary tree log-likelihood (tree+coalescent) in the posterior samples. For example, a value of 94% means that the summary tree log-likelihood was higher than 94% of the posterior trees. The CAE% column show the mean clade age errors per clade, as a percent of tree height. The CME% column shows the mean number of missed clades, as a percentage of the number of non-trivial clades in the tree. The means are obtained by averaging the statistic over the 2000 summary trees produced by each method.

## Discussion

Clearly no method in Table 1 is “best”, but several interesting trends and patterns can be identified. The agreement of ranking by comparison and magnitude is excellent, suggesting a similar distribution of errors for all methods. The table shows 22 of the 55 methods examined; most of the reduction comes from removing methods using CCD and HPF to select a topology, as MCC/TCB were significantly better for almost all combinations of methods and error criteria. This is slightly surprising, especially since we expected CCD, which assigns a proper probability to every tree topology, to fare better than heuristics such as TCB or MCC. The on-line supplement compares the four selection methods in more detail.

As expected there is a strong correlation between model fit and tree/coalescent likelihood (*r*=0.89 and *r*=0.98), but in addition the tree and coalescent likelihood are strongly correlated as well (*r*=0.85). Basically, methods generating trees with a good model fit tend to do well on both counts. The only exception is TP(avg) with a good tree likelihood but bad coalescent likelihood. Also, low clade age errors and low divergence errors go together (r=0.79), again with TP(avg) as the exception. Slightly unexpected at first sight is the negative correlation (*r*=−0.88) between clades missed and clades called. Either a method plays it safe by calling only definite clades, and tends to miss a lot (CONS), or calls everything and makes more mistakes (TP).

The table shows a second unexpected result: strong negative correlation between clade age errors and model fit (*r*=−0.94). Since model fit is highly correlated with branch lengths (*r*=0.87), no method provides good clade ages and good branch lengths/model fit. Methods optimizing branches, such as RBS, generate trees with good fit but worse ages, and methods optimizing ages exhibit the opposite. This negative correlation exists between all measures of age and fit. It is quite interesting that the two variants of the TP end up at different ends: medians give better model fit while means gives lower divergence errors.

Another performance split can be observed between pairs employing the same method for setting branch lengths but using MCC and TCB for selecting the topology. The MCC variant has better model fit, while the TCB fares better with clade calls and misses.

While Table 1 makes it easy to compare pairs of methods, it is quite hard to interpret as a whole. Table 2 complements it by aggregating some performance ranking and adding a few per-method statistics. The first statistic is the mean number of zero length branches in the summery tree, which effectively create polytomies. The methods in the table are divided into three groups: those who never create polytomies, those with occasional polytomies (up to 5%), and those with a high number (20% or more). The number of polytomies is strongly correlated with missed and called clades: methods which “resolve” conflict in the posterior by not committing and creating zero length branches miss more true clades but make less mistakes, and so have a high clade calls. Somewhat surprisingly there is no connection between zero length branches and model fit. We suspected that short branches were the main cause for low model fit, since they create non-coalescent like trees, however we see that RBS methods manage to have high model fit and around 24% polytomies. The other three statistics are the mean model fit percentile, clade age errors per clade as a percent of tree height, and the percent of missed clades from the total number of non-trivial clades. Those numbers can help in deciding how a difference in ranking translates to performance: for example, TP(avg) is seven ranks higher than TP(med) in clade time errors, but this amounts only to a difference of 0.13%, about ^1^/_7_ of the total range. On the other hand there are a seven ranks between TP(avg) and CAT in model fit, but here the difference is very large - from 45% to 93%.

**Figure 1:**
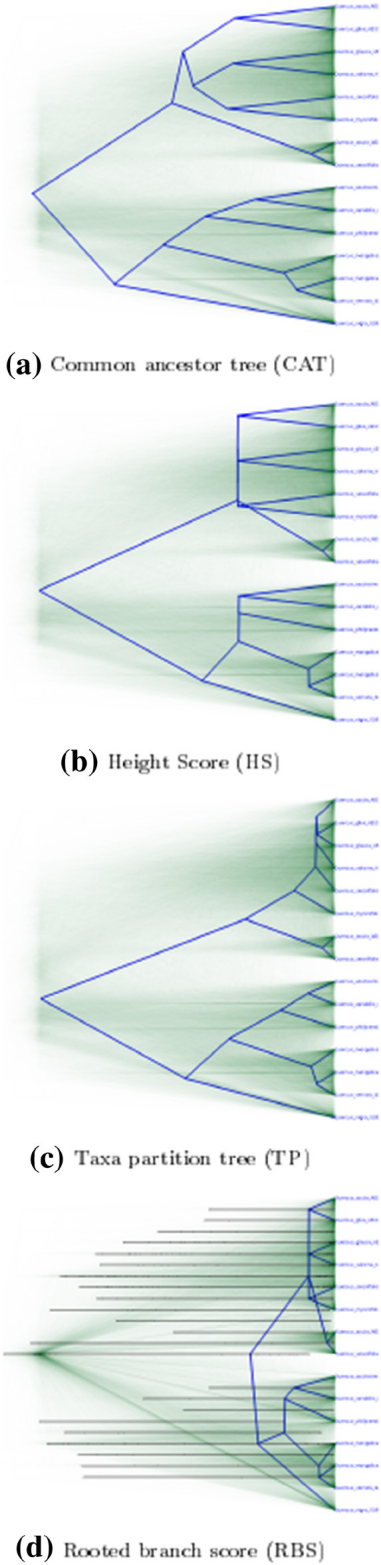
**Four Summary Trees.** Four summary trees generated from the same data set drawn over a DensiTree. A DensiTree draws all trees in a set of trees using transparancy so that in places where the trees in the tree set agrees there is dark colouring, while in places where there is a lot of variation there is light colouring. The bars in the tree from RBS shows the 95% credible intervals for all clades.

Figure 1 illustrates visually how conflict in the posterior affects the summery trees generated by four methods. The posterior trees are from a preliminary analysis of the rps16 intron of Quercus, part of a niche evolution study (Xu et al., in prep). We use this example because the weak phylogenetic signal makes the differences stand out. HS sets to zero all branches with low support, effectively creating polytomies where competing topologies exist. CAT takes the chosen topology as the truth and treats the conflicting information as “noise” to eliminate. TP is somewhere in between, and RBS creates very short branches, because a long branch for a clade with low support is penalized when the tree is matched with the many posterior trees missing the clade. Clearly RBS goes somewhat astray here, but one should keep in mind that some branches produced by other methods are unreliable too. Large discrepancy between summary trees indicates a large amount of uncertainty in the posterior, and in those cases no single tree is a good representative of the full posterior.

## Conclusions

Properly analysing the test cases proved to be as challenging as the research itself. The number of possible methods for constructing summary trees, coupled with the number of ways of assessing the results can be overwhelming. In addition, the domain of trees is vast and the evaluation and construction methods are not independent. For example, the distance between the summary and true tree seems the most natural error measure. However, we have four ways of measuring distance and four related methods, each searching for the minimal distance tree using that distance. Not unexpectedly, each distance score finds the tree produced by its counterpart to be closer to the true tree than the trees generated using other scores. Interconnections such as these show the importance of using multiple error criteria when comparing methods. The space of tree sets is complex, and each measure sheds light on different aspects of that space. Both “Clades Missing” and “Clades Called” measure topological distance via success in detecting clades, both seem reasonable and valid, yet one is the reverse of the other. Having only one of them would give a biased view. Simultaneously examining many methods – while complicating the comparison process – can reveal general performance trends.

By examining results from a large simulation study we found there is no clear “winner”. Having low clade age errors and good branch lengths in a tree seems fundamentally exclusive. Methods setting clade ages from posterior ages tend to have lower age errors while methods matching the branch lengths produce trees with a better fit to the model.

Therefore, it makes sense to consider the purpose of the summary tree when choosing a method. If divergence times matters most, use either HS or CAT. If only topology matters, use the consensus (CONS) or TP. In both cases the decision between the two alternatives depend on whether you are conservative and prefer unresolved clades (polytomies) in areas of conflict, or whether you wish a “the best guess” at a fully resolved tree. Use RBS to get a tree with good model fit and therefore closer to a Maximum Likelihood tree.

TP(med) provides a good compromise: good model fit and low missed clades, with middle of the pack ages/divergence errors (but still better than RBS). All of these are better than the MCC as implemented in TreeAnnotator, which is middle of the pack in all measures except for doing worse on clades called and well on root height.

While the simulations show a few surprising results, we were most surprised by the performance of the “theory based” methods. We set out to replace heuristics with methods based upon firmer theoretical consideration, and strongly expected that RAS, a tree metric which takes into account both ages and branch lengths, will outperform the alternatives. Likewise, we expected the CCD to fare better than other methods for selecting a topology. However, heuristics seem to do better when measured against the main objective - recovering the true tree.

We think the different types of summaries are all valuable when the posterior trees are in conflict. Together with the full posterior as drawn by DensiTree, they provide different insights into the information contained in the posterior. We suggest that researchers generating a summary tree for annotation or publication use one of the newer methods since all of them outperform the existing consensus methods and BEAST’s own TreeAnnotator.

While we focused on obtaining a single point estimate from posterior MCMC samples, we would like to emphasize that researchers should treat single point estimates as end points, and use the full posterior whenever possible, especially for secondary analyses. In addition, one should look at several methods for extracting a point estimate when dealing with the complex space of phylogenetic trees.

## Appendix

### A Taxa partitions formal definition

Formally, For taxa ordering $L=(x_{1},x_{2},\ldots )$ and clade *c*, let $I=(i_{1},i_{2},\ldots )$ be the set of ordered indices of *c* in *L*, that is $c=(x_{i_{1}},x_{i_{2}},\ldots )$ and *i*_1_<*i*_2_<…<*i*_|*c*|_. The span of the clade is the range of consecutive integers covering *I*,

$$\mathrm{sp}(c,L)=(i_{1},i_{1}+1,i_{1}+2,\ldots ,i_{\left|c\right|}).$$

Now, *c* is compatible with *L* at position *k* if

$$\begin{array}{l} \mathrm{compat}(c,L,k)\equiv \;\;\;\;\;\;\;\;\;|c|=|\mathrm{sp}(c,L)|\\ \;\text{and}\;\;\;k\in \mathrm{sp}(c,L)\\ \;\text{and}\;\;\;\mathrm{sp}(S_{1}(c),L)\cap \mathrm{sp}(S_{2}(c),L)=\emptyset \end{array}$$

where *S*_1,2_(*c*) are the left and right sons of *c*.

The contribution for the k’th split comes from all trees containing a compatible clade at this point,

$$\;V_{k}=\left\{h(C):(L,\mathbb{C},h)\in T\text{and}C\in \mathbb{C}\text{and}\mathrm{compat}(c,L,k)\right\}$$

The ages are computed by taking the median (or mean) of *V*_*k*_. The tree is reconstructed by picking the maximal age as the root, and recursively building the sub-trees to the left and right of the split.

## B Implementation: Minimum distance tree

For a tree set  (draws from the posterior) and a target topology  our objective is to find a tree with topology  which minimizes the total distance to  under a tree distance metric *d*_*T*_ (Equation 13). We use a generic multivariate optimizer to do part of the heavy lifting, but transforming the problem into a suitable form is far from trivial. While the details vary slightly for each distance metric, we found the following four steps essential: 

1. Represent the tree as a point in ${\mathbb{R}}_{m}$.

2. Pre-process posterior trees to speed up the evaluation of the total distance to all posterior trees.

3. Analytically compute the derivative.

4. Find a good initial starting point.

### Tree parametrization

The tree T=(L,ℂ,h) is represented as a vector of real numbers $z=(h_{r},\alpha_{2},\ldots ,\alpha_{m})\in {\mathbb{R}}_{m}$, where m=|ℂ|−|L| is the number of internal clades in the tree. *h*_*r*_ is the height of *T*, and $\alpha_{2},\alpha_{3},\ldots ,\alpha_{m}$ are *m*−1 values, one per internal clade, equal to the ratio of the clade age to the age of its parent. To retrieve a clade age from *z*, multiply the root height by the *α* for all the clade ancestors. That is, $h_{r}\underset{k}{\prod }\alpha_{k}$, where *k* ranges over the clade ancestors. By traversing the tree in pre-order (clade before its descendants) all ages can be extracted from *z* using just *m*−1 multiplications, and an additional 2(*m*−1) subtractions would extract all branch lengths. Each component in *z* has a simple bound independent of other components; 0≤*h*_*r*_<*∞* and 0≤*α*_*i*_≤1. This makes the tree a suitable optimization target for a method such as L-BGFS-B, a quasi newton algorithm for minimizing a multivariate function with simple bounds [18].

### Pre-processing of posterior trees

The search for the minimum distance tree involves many evaluations of the target function, the mean distance d(T,T). This evaluation is sped up by transforming the expression, which is a sum on trees, into a sum over clades. The details vary somewhat, depending on the distance metric *d*_*T*_. Here we elaborate for the rooted branch score case (Equation 8), and the interested reader should consult the code for details of the other metrics.

For the tree T=(L,ℂ,h) the total distance is expanded as follows,

$$\begin{array}{ll} \;d(T,T)=\underset{t_{i}\in T}{\sum }\text{RBS}(T,t_{i})\; & \;\\ \;=\underset{t_{i}\in T}{\sum }\underset{x\in {\mathbb{C}}_{i}\cup \mathbb{C}}{\sum }|b_{i}(x)-b(x)|\; & \;\\ \;=\underset{t_{i}\in T}{\sum }\left[\underset{x\in {\mathbb{C}}_{i}\cap \mathbb{C}}{\sum }\;|b_{i}(x)-\;b(x)|+\;\;\;\underset{x\in \mathbb{C}\setminus {\mathbb{C}}_{i}}{\sum }b(x)+\;\;\;\;\underset{x\in {\mathbb{C}}_{i}\setminus \mathbb{C}}{\sum }b_{i}(x)\right]\; & \;\\ \;=\underset{t_{i}\in T}{\sum }\underset{x\in {\mathbb{C}}_{i}\cap \mathbb{C}}{\sum }|b_{i}(x)-b(x)|+\underset{t_{i}\in T}{\sum }\underset{x\in \mathbb{C}\setminus {\mathbb{C}}_{i}}{\sum }b(x)\; & \;\\ \;+\underset{t_{i}\in T}{\sum }\underset{x\in {\mathbb{C}}_{i}\setminus \mathbb{C}}{\sum }b_{i}(x)\; & \;\\ \;=\underset{t_{i}\in T}{\sum }\underset{x\in {\mathbb{C}}_{i}\cap \mathbb{C}}{\sum }|b_{i}(x)-b(x)|+\underset{x\in \mathbb{C}}{\sum }b(x)\left(\underset{t_{i}\in T}{\sum }x\notin {\mathbb{C}}_{i}\right)\; & \;\\ \;+\left(\underset{t_{i}\in T}{\sum }\underset{x\in {\mathbb{C}}_{i}\setminus \mathbb{C}}{\sum }b_{i}(x)\right).\; & \; \end{array}$$

The terms in parentheses do not depend on *T* and can be precomputed, so the last two terms take O(|ℂ|) operations to evaluate. The first term appears to require O(|ℂ||T|) but we can cut this down to O(|ℂ|log(|T|)).

$$\begin{array}{lll} & \underset{t_{i}\in T}{\sum }\underset{x\in {\mathbb{C}}_{i}\cap \mathbb{C}}{\sum }|b_{i}(x)-b(x)|=\underset{x\in \mathbb{C}}{\sum }\underset{\begin{array}{c} t_{i}\in T\\ x\in {\mathbb{C}}_{i} \end{array}}{\sum }|b_{i}(x)-b(x)|\; & \;\\ = & \underset{x\in \mathbb{C}}{\sum }\left[\underset{\begin{array}{c} t_{i}\in T\\ x\in {\mathbb{C}}_{i}\\ b(x)>b_{i}(x) \end{array}}{\sum }b(x)-b_{i}(x)+\underset{\begin{array}{c} t_{i}\in T\\ x\in {\mathbb{C}}_{i}\\ b(x)\leq b_{i}(x) \end{array}}{\sum }-b(x)+b_{i}(x)\right]\; & \;\\ = & \underset{x\in \mathbb{C}}{\sum }\left[\left(\underset{\begin{array}{c} t_{i}\in T\\ x\in {\mathbb{C}}_{i}\\ b(x)>b_{i}(x) \end{array}}{\sum }1-\underset{\begin{array}{c} t_{i}\in T\\ x\in {\mathbb{C}}_{i}\\ b(x)\leq b_{i}(x) \end{array}}{\sum }1\right)b(x)\right)\; & \;\\ & \left(-\left(\underset{\begin{array}{c} t_{i}\in T\\ x\in {\mathbb{C}}_{i}\\ b(x)>b_{i}(x) \end{array}}{\sum }b_{i}(x)\right)+\left(\underset{\begin{array}{c} t_{i}\in T\\ x\in {\mathbb{C}}_{i}\\ b(x)\leq b_{i}(x) \end{array}}{\sum }b_{i}(x)\right)\right]\; & \; \end{array}$$

The reason for this complicated looking expression is that the last two terms in parentheses can be pre-computed, and the first is simply the number of branches in the posterior greater than *b*(*x*) minus the number of branches smaller than it. After we pre-sort the branches from the posterior for each clade, this number can be found by a binary search, taking at most O(log(|T|)) since there can be at most (|T|) matched branches, one for each tree in the posterior.

### Analytical derivative

The search is significantly faster when a derivative can be computed analytically, since estimating a derivative requires at least *m* evaluations (the number of dimensions). While the details are tedious the calculations themselves are simple, since the target function is composed in a series of multiplications and additions/subtractions, so the derivative is easy to compute using the chain rule at each stage. Again the interested reader should consult the code for the exact details in each case.

### Search initialization

We found that a good starting point can be vital, as under some settings the number of multiple local minima can be large. While the procedure to obtain the initial tree seems natural and obvious in hindsight, several other obvious looking approaches did not perform well at all.

The initial tree is obtained by first examining each branch independently. Each branch has its own optimal length, based on the matching branches in the posterior and the distance metric. This optimal value is computed for each branch, but since branch lengths are not independent, the next step builds a tree from those optimal values. The build assigns an age to each clade, proceeding in post-order, that is assigning an age to all descendants of a clade before assigning the clade age. The age of the clade is obtained by averaging the expected age from the direct descendants, which is the sum of their own assigned age and their optimal branch length.

## Abbreviations

AVG: Summary tree method which sets internal nodes heights using average of posterior heights; ca(): Common ancestor; CAE: Clades ages error. Equation (1); CAT: Common ancestor tree. Equation (14); CC: Clade credibility; CCD: Conditional clade probability distribution; CCE: Clades called error. Equation (6); CLL: Coalescent likelihood; CME: Clades missed error. Equation (5); DVE: Divergence times error. Equation (2); HS: Heights score. Equation (10); HSO: Heights only summary tree method; HPF: Highest posterior frequency; MCC: Maximal clade credibility; MED: Summary tree method which sets internal nodes heights using medians of posterior heights; RAS: Rooted agreement score. Equation (11); RBS: Rooted branch score. Equation (8); RH: Root height; SRBS: Squared rooted branch score. Equation (9); TCB: Total clade branch; TP: Taxa partition tree.

## Competing interests

The authors declare that they have no competing interests.

## Authors’ contributions

JH developed the methods and wrote the biopy implementation. RB Implemented the DensiTree integration. Both authors contributed to the manuscript. Both authors read and approved the final manuscript.

## Supplementary Material

Additional file 1 **Supplementary material.** Information about lesser performing methods which are mentioned only briefly in the main text. Click here for file

Additional file 2 **Posterior summary rank graphs.** Method rank graphs for each error measure. Click here for file
