# Supplementary material for: Looking for trees in the forest: summary tree from posterior samples
Source: BMC Evol Biol. 2013 Oct 4;13:221. doi: 10.1186/1471-2148-13-221 (PMC3853548; doi:10.1186/1471-2148-13-221)
Supplement: Additional file 1 — Supplementary material. Information about lesser performing methods which are mentioned only briefly in the main text. [file 1471-2148-13-221-S1.PDF]

Summary Tree from Posterior Samples: Supplemental Information

Joseph Heled

December 4, 2012

Selecting a Topology

We examined five methods for selecting the of the summary tree topology, MCC, TCB, CCD, HPF and HPV. The first four are explained in the article, and the last is simply picking the topology of the tree with the highest posterior value. The article shows results for only for MCC and TCB, and here we show why. Table 1 and 2 summarise the pairwise performance of the five methods. Each method can be coupled with one of the 7 ways of assigning branch lengths, RBS,SRBS,HSO,RAS,MED,CAT, and AVG. For each test case there are 7 pairing: for example, when comparing MCC and TCB we have MED,TCB Vs. MED,MCC, RBS,TCB Vs. RBS,MCC, HSO,TCB Vs. HSO,MCC and so on. For each pairing there are 8 error measure. Table 1 shows the percentage of cases where method X was significantly better than Y, computed over the all pairings, error measures and 4000 bootstrap trials.

Table 1

	MCC	TCB	CCD	HPF	HPV
MCC		0.51	0.83	0.86	0.91
TCB	0.49		0.84	0.88	0.9
CCP	0.17	0.16		0.74	0.88
HPF	0.14	0.12	0.26		0.93
HPV	0.094	0.1	0.12	0.068	

Table 2 shows the number of times method X was significantly better than Y, summed over all 7×8 branch assignment methods and error measure combinations.

Table 2

	MCC	TCB	CCP	HPF	HPV
MCC		23	44	44	47
TCB	21		46	46	49
CCP	6	6		35	43
HPF	4	4	9		48
HPV	4	3	5	2	

The tables clearly show that MCC and TCB are evenly matched, and are significantly better than the other 3.

Full rank tables

Table 3 is identical to the ranking table in the article, with an additional 4 error measures. The four are the distance of the summary tree to the true tree under each distance measures.

Table 3: See main article for the description of table columns.

method	RH	CM	CC	CH	DV	MF	TLL	CLL	dHS	dSRBS	dRBS	dRAS
TP (med)	1/3	0/0	12/9	8/8	7/5	3/3	0/0	3/3	7/7	4/7	7/9	2/2
mRBS	6/8	11/11	3/3	13/13	11/10	0/0	2/1	2/2	11/11	4/3	0/0	6/3
TP (avg)	0/4	0/0	13/9	6/7	0/3	11/10	1/6	14/15	7/8	3/6	8/10	3/4
MED,TCB	1/0	3/3	10/7	6/4	6/6	9/7	8/11	9/9	14/15	8/11	9/12	10/6
RBS,TCB	6/8	10/10	4/3	12/12	11/10	2/2	4/3	1/1	10/10	5/4	1/1	7/4
RBS,MCC	7/9	12/12	5/4	12/12	11/11	1/1	3/2	0/0	11/12	6/5	2/2	8/5
HSO,TCB	1/1	2/2	11/8	6/4	6/6	10/7	8/11	10/10	14/15	8/11	9/11	10/6
MED,MCC	1/0	6/6	12/10	7/4	7/7	7/6	7/10	7/7	15/16	10/12	10/14	11/7
HSO,MCC	1/2	5/5	13/11	6/4	7/7	8/6	7/10	8/8	15/16	10/12	10/13	11/7
SRBS,TCB	3/5	8/7	8/5	6/5	1/2	12/9	6/9	13/13	8/9	1/1	6/7	4/4
RAS,MCC	5/6	14/14	7/4	9/9	9/8	4/4	3/4	6/6	5/5	7/9	3/4	1/1
mSRBS	3/5	9/8	9/6	7/6	2/3	11/8	4/8	12/11	8/9	0/0	5/6	3/3
RAS,TCB	5/6	13/13	6/3	10/10	10/9	5/4	5/5	5/5	4/4	7/8	3/3	1/1
SRBS,MCC	4/6	9/9	9/6	6/5	3/4	11/9	5/9	11/12	9/10	2/2	6/8	5/5
mRAS	5/7	15/15	7/4	11/11	10/9	6/5	6/7	4/4	6/6	7/10	4/5	0/0
mHS	1/0	18/19	1/1	2/0	1/0	16/12	12/14	18/18	0/0	15/15	13/17	13/10
AVG,MCC	0/4	7/7	11/9	5/5	5/8	13/11	9/12	15/14	16/17	9/12	12/15	12/8
CAT,TCB	0/5	1/1	14/10	0/0	1/2	18/16	14/15	20/21	12/13	11/13	13/17	14/11
HS,MCC	1/1	19/18	2/2	3/2	3/0	14/13	10/16	17/17	2/2	14/14	11/16	9/9
CAT,MCC	0/4	4/4	15/12	1/1	1/2	17/15	13/14	19/20	13/14	12/14	14/18	14/11
HS,TCB	2/2	17/17	2/2	4/3	4/1	15/14	11/17	16/16	1/1	13/13	11/16	9/9
CONS (med)	1/0	16/16	0/0	3/2	8/8	17/14	15/13	19/19	3/3	16/16	15/18	15/11

Table 4 has the same columns as table 3, and includes all tested methods.

Table 4: See main article for the description of table columns.

method	RH	CM	CC	CH	DV	MF	TLL	CLL	dHS	dSRBS	dRBS	dRAS
TP (med)	1/4	0/0	23/17	15/10	9/6	7/8	0/0	7/7	13/13	7/9	15/17	6/6
MED,TCB	2/0	3/3	21/15	7/5	8/7	16/15	11/16	18/17	29/31	18/15	23/25	23/20
HSO,TCB	1/1	2/2	22/16	7/5	8/7	17/15	11/16	19/18	29/31	18/15	23/24	23/20
MED,MCC	2/0	6/6	23/18	8/5	9/8	14/14	10/15	16/15	30/32	20/16	24/27	24/21
HSO,MCC	1/2	5/5	24/19	8/5	9/8	15/14	10/15	17/16	30/32	20/16	24/26	24/21
RBS,mSRBS	9/14	24/22	8/9	24/19	20/17	1/1	4/2	6/6	18/18	7/4	0/0	12/9
RBS,mRBS	10/15	25/23	7/8	25/20	21/18	0/0	3/1	5/5	19/19	8/5	0/0	13/10
RBS,TCB	10/15	22/21	8/8	24/18	21/17	3/3	6/4	4/4	17/17	9/6	1/2	15/12
RAS,mRBS	6/8	28/27	11/8	18/13	13/10	9/9	1/1	13/13	9/10	9/11	7/7	1/1
RBS,mHS	11/15	23/22	6/7	23/18	21/18	4/4	6/5	4/3	16/16	10/6	1/1	14/11
TP (avg)	0/5	0/0	24/17	8/8	0/3	23/23	2/9	35/36	13/14	6/8	19/20	8/10
RBS,MCC	12/16	26/24	9/10	23/18	21/18	2/2	5/3	3/3	19/20	11/7	2/3	16/13
RAS,mSRBS	6/8	29/27	13/10	18/13	12/9	10/10	2/3	15/14	9/10	8/10	8/7	1/1
SRBS,TCB	4/6	8/7	19/13	8/6	1/2	24/22	8/13	34/34	15/15	1/1	14/15	9/10
SRBS,mSRBS	4/6	9/8	20/14	9/7	2/3	23/21	6/12	33/32	15/15	0/0	13/14	8/9
RAS,mHS	6/8	29/28	9/7	19/14	15/12	12/11	8/7	14/12	7/8	13/12	9/8	2/2
RAS,MCC	7/9	31/29	13/11	18/13	14/11	11/11	5/6	13/11	9/10	12/13	9/10	1/2
SRBS,mHS	5/7	13/12	17/13	13/9	3/3	20/20	8/13	29/30	13/14	2/2	11/12	7/8
SRBS,MCC	5/8	9/9	20/14	8/6	3/4	23/22	7/13	32/33	16/17	2/2	14/16	10/11
RAS,TCB	7/9	30/28	12/9	19/14	15/12	12/12	7/8	12/10	8/9	12/12	9/9	1/1
RBS,mRAS	13/18	27/26	8/8	26/21	22/19	5/5	6/6	5/3	20/21	12/8	3/4	11/8
MED,CCD	1/0	12/11	25/20	10/8	12/12	21/16	16/18	19/19	32/34	24/19	27/29	27/23
SRBS,mRBS	7/9	17/16	17/14	14/11	6/5	18/19	5/11	26/28	15/16	3/3	11/12	8/9
HSO,CCD	1/1	11/10	26/21	10/8	12/12	22/16	16/18	20/20	32/34	24/19	27/29	27/23
RBS,CCD	12/17	28/25	9/8	27/22	23/20	7/6	10/9	2/2	21/22	14/8	4/5	18/14
RAS,mRAS	6/10	32/30	14/10	20/15	15/12	13/13	8/10	11/9	10/11	12/14	10/11	0/0
SRBS,mRAS	8/11	14/16	20/20	13/10	4/2	19/20	6/14	27/27	15/16	3/4	12/13	7/7
RBS,HPF	13/17	33/29	9/7	28/23	24/21	6/5	11/10	1/1	23/24	15/9	5/5	19/15
AVG,MCC	0/5	7/7	22/17	6/6	6/10	31/26	15/19	36/35	31/33	19/16	26/28	26/22
MED,HPF	1/0	16/15	28/23	10/8	11/11	27/18	18/20	21/21	33/34	25/19	29/29	29/23
SRBS,CCD	6/9	14/13	18/13	12/9	7/6	27/24	9/17	31/33	21/23	4/4	16/18	13/16
HSO,HPF	2/2	15/14	29/24	10/8	11/11	28/18	18/20	22/22	33/34	25/19	28/29	28/23
CAT,TCB	0/6	1/1	26/18	0/0	1/2	40/31	21/23	42/44	25/27	25/20	31/33	31/27
RAS,CCD	7/11	34/32	15/10	21/16	18/15	17/14	12/18	8/8	11/12	16/15	11/12	3/3
CAT,MCC	0/5	4/4	27/20	1/1	1/2	39/30	20/22	41/43	26/28	26/21	32/34	31/27
SRBS,HPF	7/9	20/17	18/14	14/11	8/7	25/23	10/17	29/31	22/24	5/4	17/17	17/17
RBS,HPO	14/19	34/33	10/8	29/24	25/22	8/7	13/14	0/0	24/26	16/14	6/6	20/16
HS,mHS	1/0	36/36	1/1	3/0	1/0	37/27	19/22	40/41	0/0	33/27	31/33	30/26
RAS,HPF	7/10	38/34	15/9	22/17	18/15	18/15	14/19	9/8	12/13	17/15	12/13	4/4
HS,MCC	1/1	37/35	2/2	4/2	3/0	32/28	17/28	38/38	2/2	28/21	25/30	22/24
HS,TCB	2/2	35/34	2/2	5/3	5/1	33/29	18/29	37/37	1/1	27/20	25/30	22/23
SRBS,HPO	9/13	21/20	19/19	15/12	10/8	26/25	12/18	28/29	23/25	7/9	18/19	18/18
CONS (med)	1/0	33/31	0/0	4/2	10/9	39/29	23/21	41/42	5/3	34/28	36/38	33/29
MED,HPO	2/0	19/19	29/26	10/8	12/13	29/25	22/26	24/23	34/35	29/22	30/30	30/24
RAS,HPO	8/12	39/38	16/12	23/18	19/16	19/17	16/20	10/8	14/16	18/16	14/15	5/5
CAT,CCD	0/5	10/9	29/22	1/1	3/5	41/32	23/24	43/45	28/31	28/23	33/35	31/26
HSO,HPO	2/2	18/18	30/27	11/8	12/13	30/25	22/26	25/24	34/35	29/22	30/30	30/24
HS,CCD	1/1	40/38	4/4	10/5	11/9	34/33	20/32	30/36	3/5	29/22	24/29	21/22
HS,HPF	1/1	41/39	3/3	7/4	9/6	37/30	21/30	37/39	3/4	31/23	28/31	25/24
CAT,HPF	0/5	14/13	31/25	0/0	3/4	42/33	24/25	44/46	27/29	30/24	34/36	32/27
HS,mSRBS	2/3	40/37	5/4	13/8	11/9	35/36	21/33	26/29	4/6	21/16	22/23	18/19
HS,HPO	1/1	43/42	4/5	8/4	10/7	38/34	23/31	39/40	5/7	32/25	30/32	26/25
CAT,HPO	0/5	17/17	32/28	2/0	5/6	43/35	25/27	45/47	28/30	33/26	35/37	33/28
HS,mRBS	2/3	42/40	8/5	16/12	16/14	36/37	22/34	24/26	6/8	22/17	20/21	15/17
HS,mRAS	3/4	43/41	9/6	17/13	17/15	36/37	22/34	23/25	7/9	23/18	21/22	14/16

Rank graphs

When focusing on a single error measure, a graphical representation may be useful too. Figure 1 shows two cases. All graphs are in the `posteriorSummaryRankGraphs.tar.gz` file.

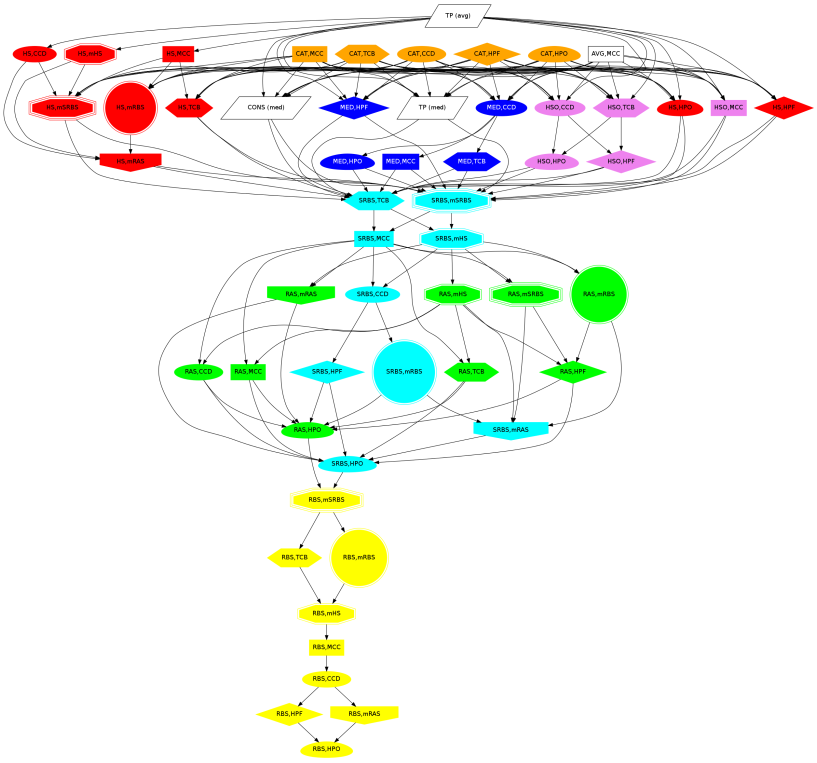

(a) Better Root Height

(b) Better Model Fit

Figure 1: Two ranking graphs. An arrows from X to Y means that X is significantly better than Y. Methods for setting branch lengths are coded by color, and topology selection methods are coded by shape.
