# Supplementary figures and images for: Looking for trees in the forest: summary tree from posterior samples
Source: BMC Evol Biol. 2013 Oct 4;13:221. doi: 10.1186/1471-2148-13-221 (PMC3853548; doi:10.1186/1471-2148-13-221)

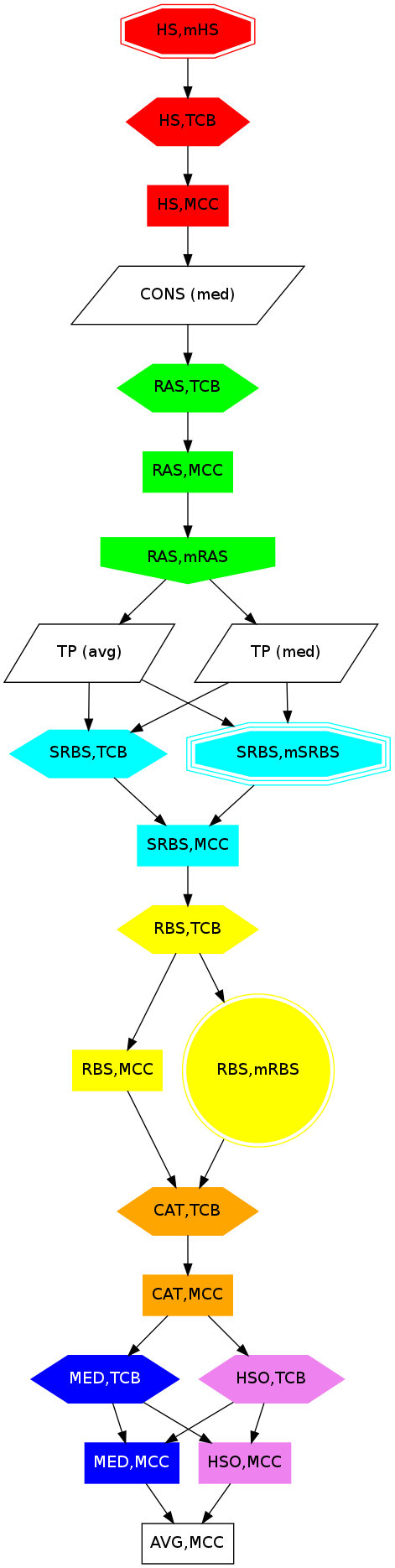

Supplement: Additional file 2 — Posterior summary rank graphs. Method rank graphs for each error measure. [file 1471-2148-13-221-S2.GZ › posteriorSummaryRankGraphs/partial/closer2truthhs.png]

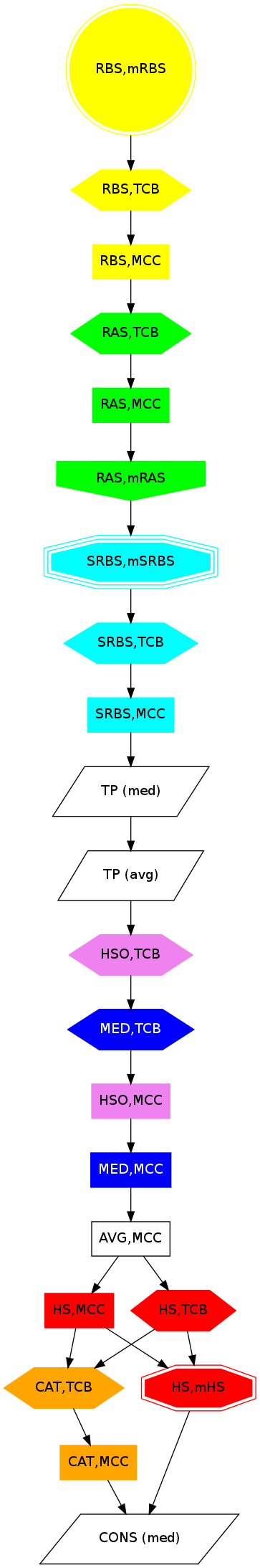

Supplement: Additional file 2 — Posterior summary rank graphs. Method rank graphs for each error measure. [file 1471-2148-13-221-S2.GZ › posteriorSummaryRankGraphs/partial/closer2truthbs1V.png]

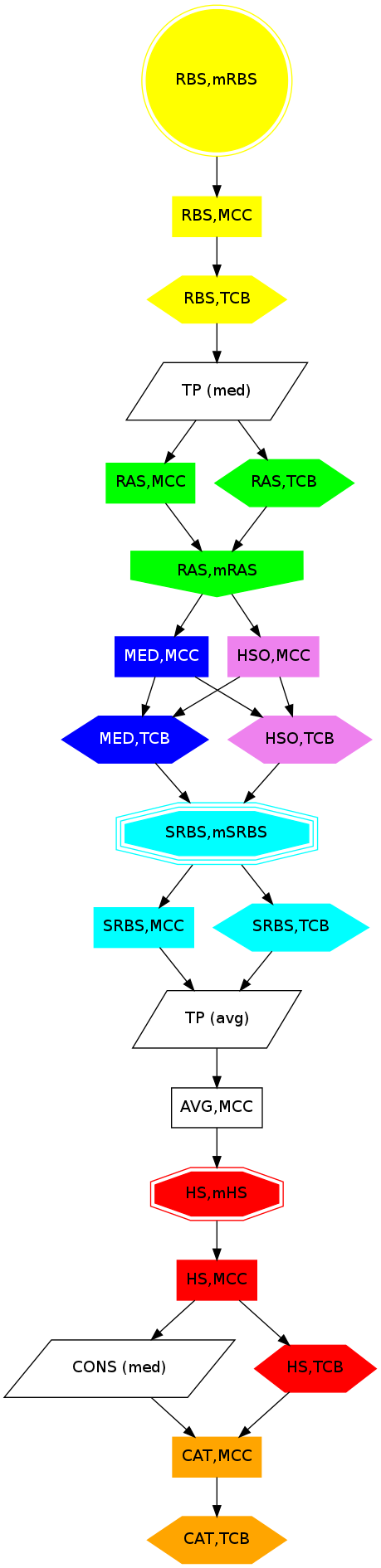

Supplement: Additional file 2 — Posterior summary rank graphs. Method rank graphs for each error measure. [file 1471-2148-13-221-S2.GZ › posteriorSummaryRankGraphs/partial/betterLLmodelV.png]

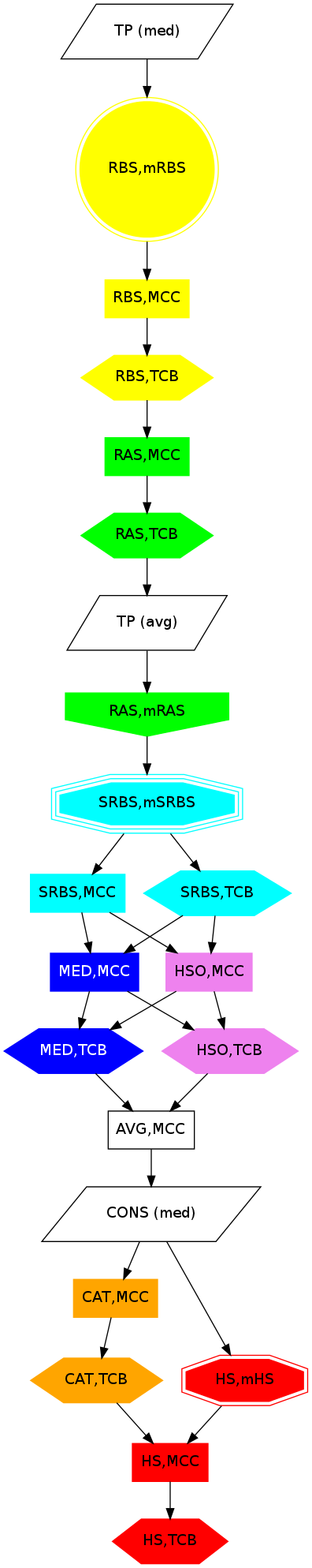

Supplement: Additional file 2 — Posterior summary rank graphs. Method rank graphs for each error measure. [file 1471-2148-13-221-S2.GZ › posteriorSummaryRankGraphs/partial/betterLLtreeV.png]

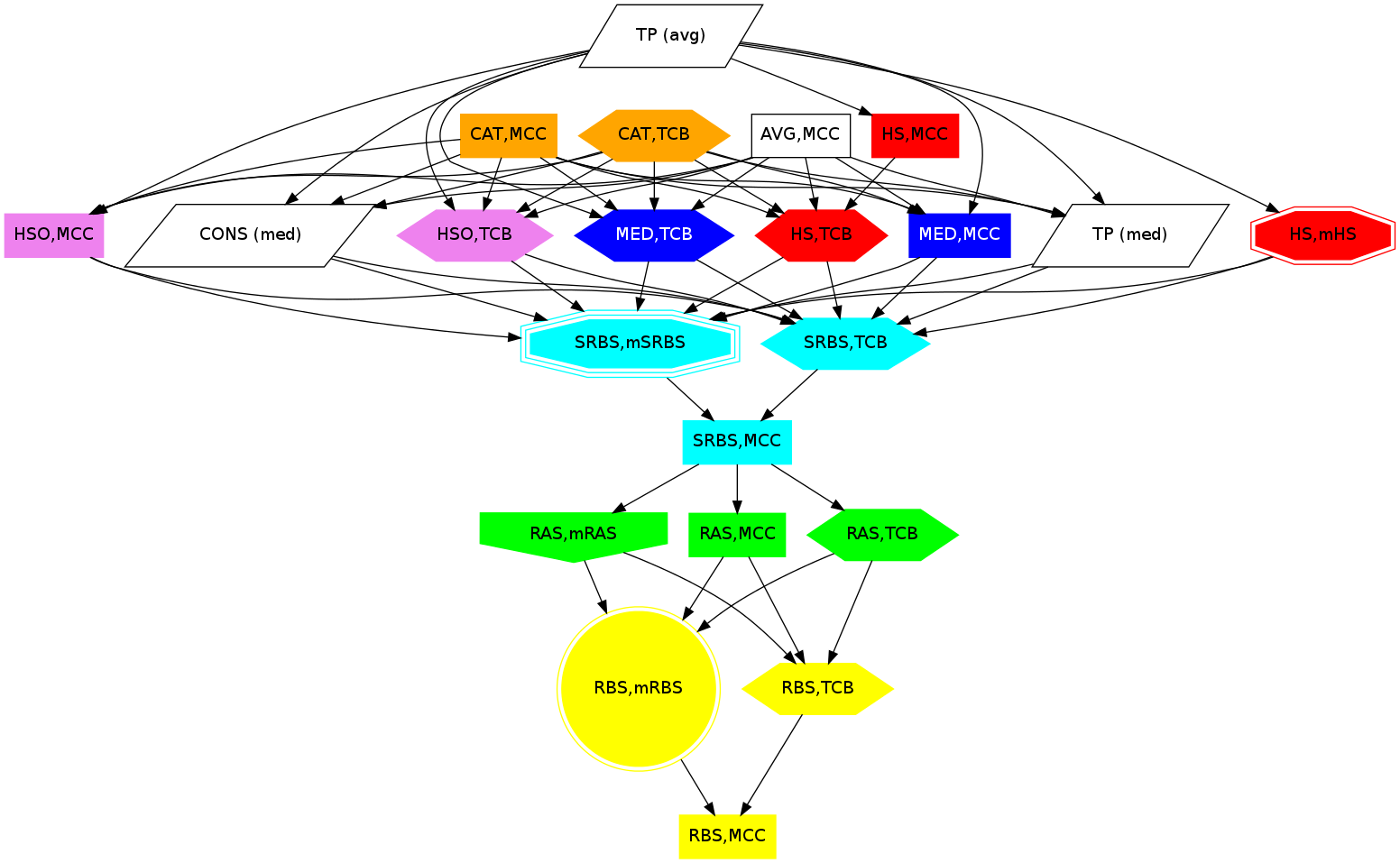

Supplement: Additional file 2 — Posterior summary rank graphs. Method rank graphs for each error measure. [file 1471-2148-13-221-S2.GZ › posteriorSummaryRankGraphs/partial/betterTimesRoot.png]

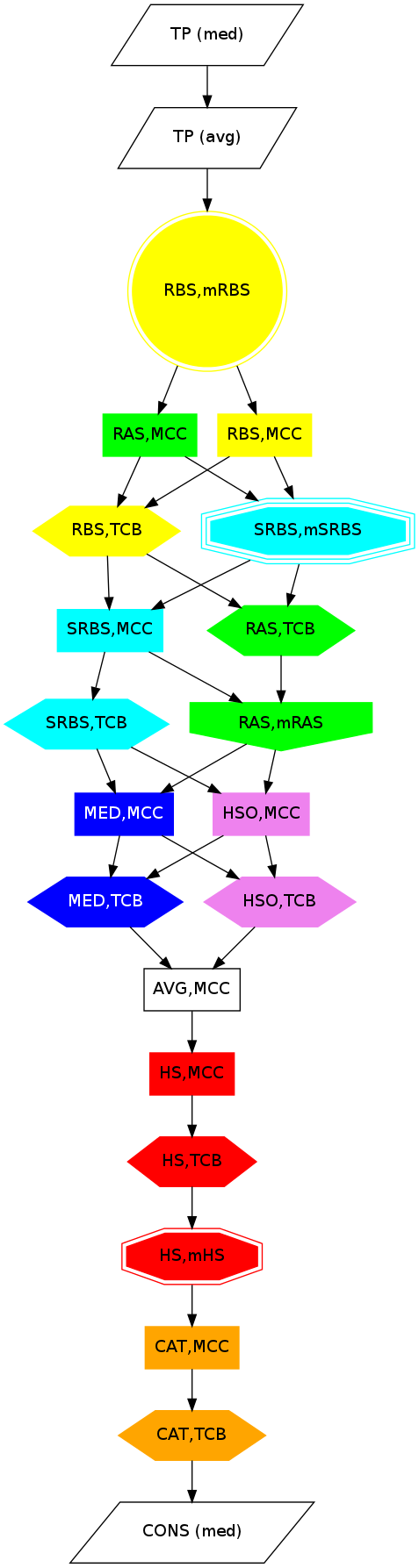

Supplement: Additional file 2 — Posterior summary rank graphs. Method rank graphs for each error measure. [file 1471-2148-13-221-S2.GZ › posteriorSummaryRankGraphs/partial/betterLLtree.png]

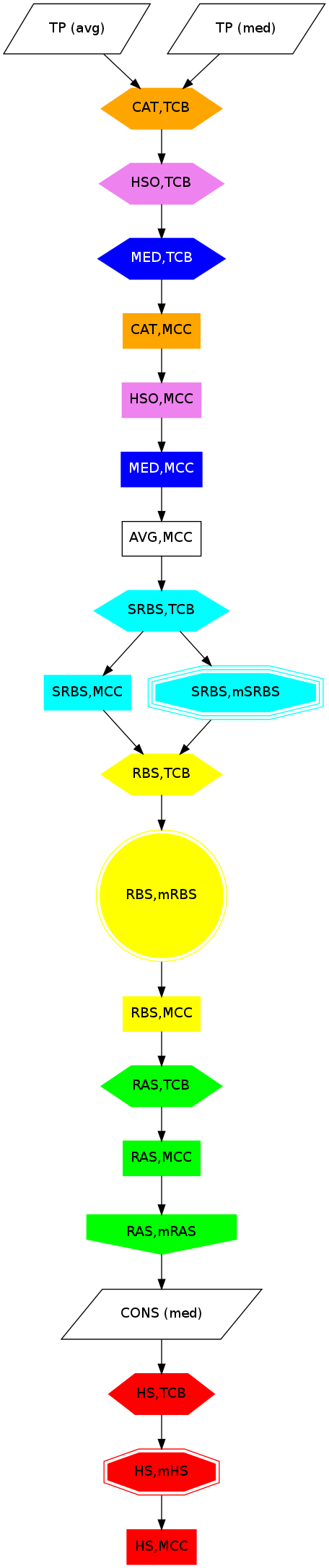

Supplement: Additional file 2 — Posterior summary rank graphs. Method rank graphs for each error measure. [file 1471-2148-13-221-S2.GZ › posteriorSummaryRankGraphs/partial/betterCladeMisses.png]

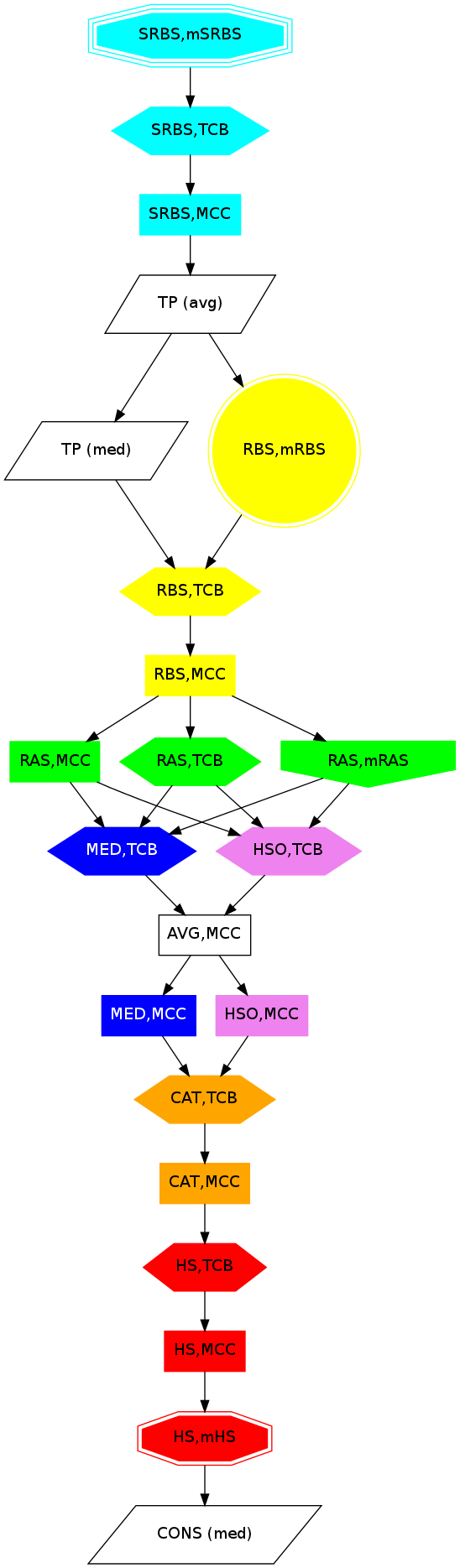

Supplement: Additional file 2 — Posterior summary rank graphs. Method rank graphs for each error measure. [file 1471-2148-13-221-S2.GZ › posteriorSummaryRankGraphs/partial/closer2truthbs2.png]

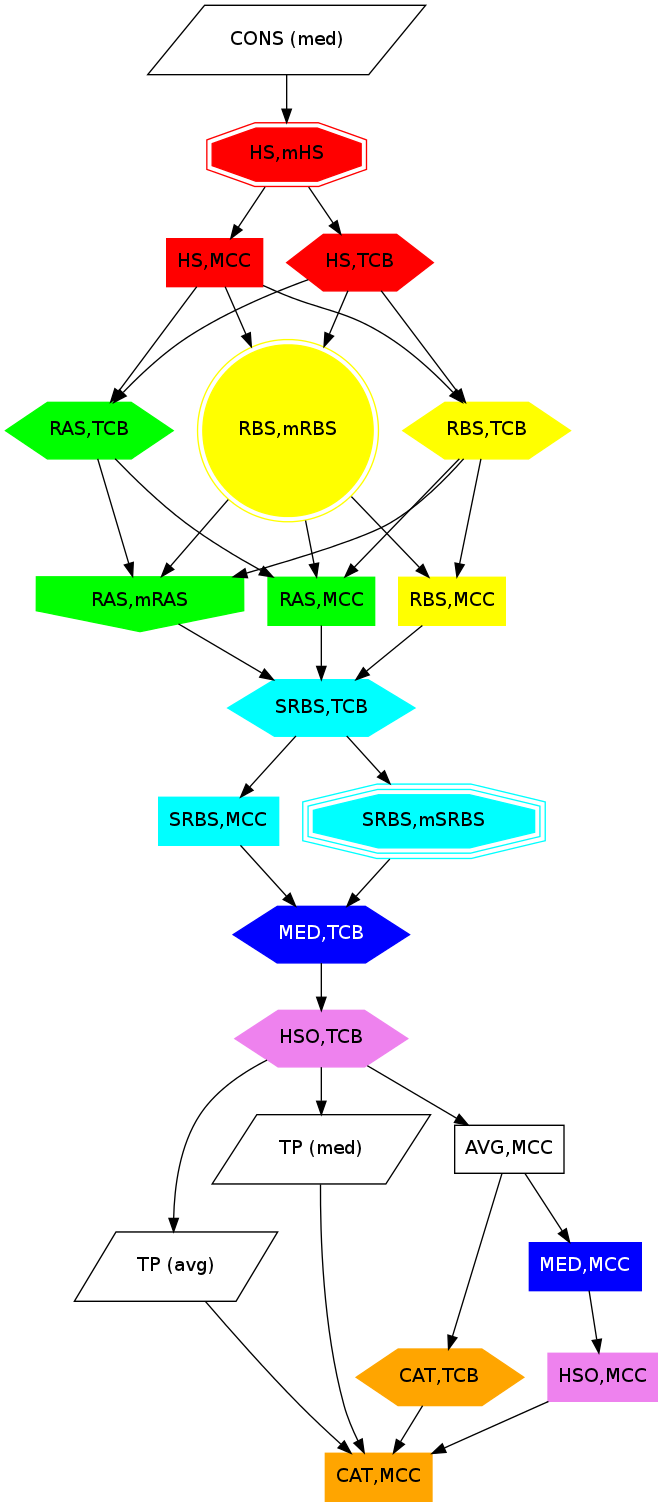

Supplement: Additional file 2 — Posterior summary rank graphs. Method rank graphs for each error measure. [file 1471-2148-13-221-S2.GZ › posteriorSummaryRankGraphs/partial/betterCladeCallsV.png]

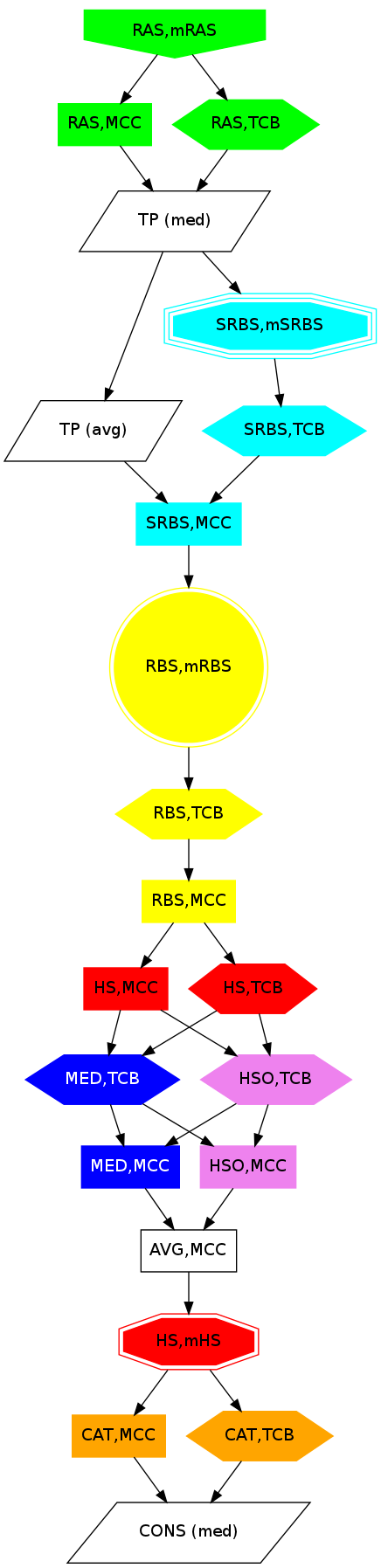

Supplement: Additional file 2 — Posterior summary rank graphs. Method rank graphs for each error measure. [file 1471-2148-13-221-S2.GZ › posteriorSummaryRankGraphs/partial/closer2truthras.png]

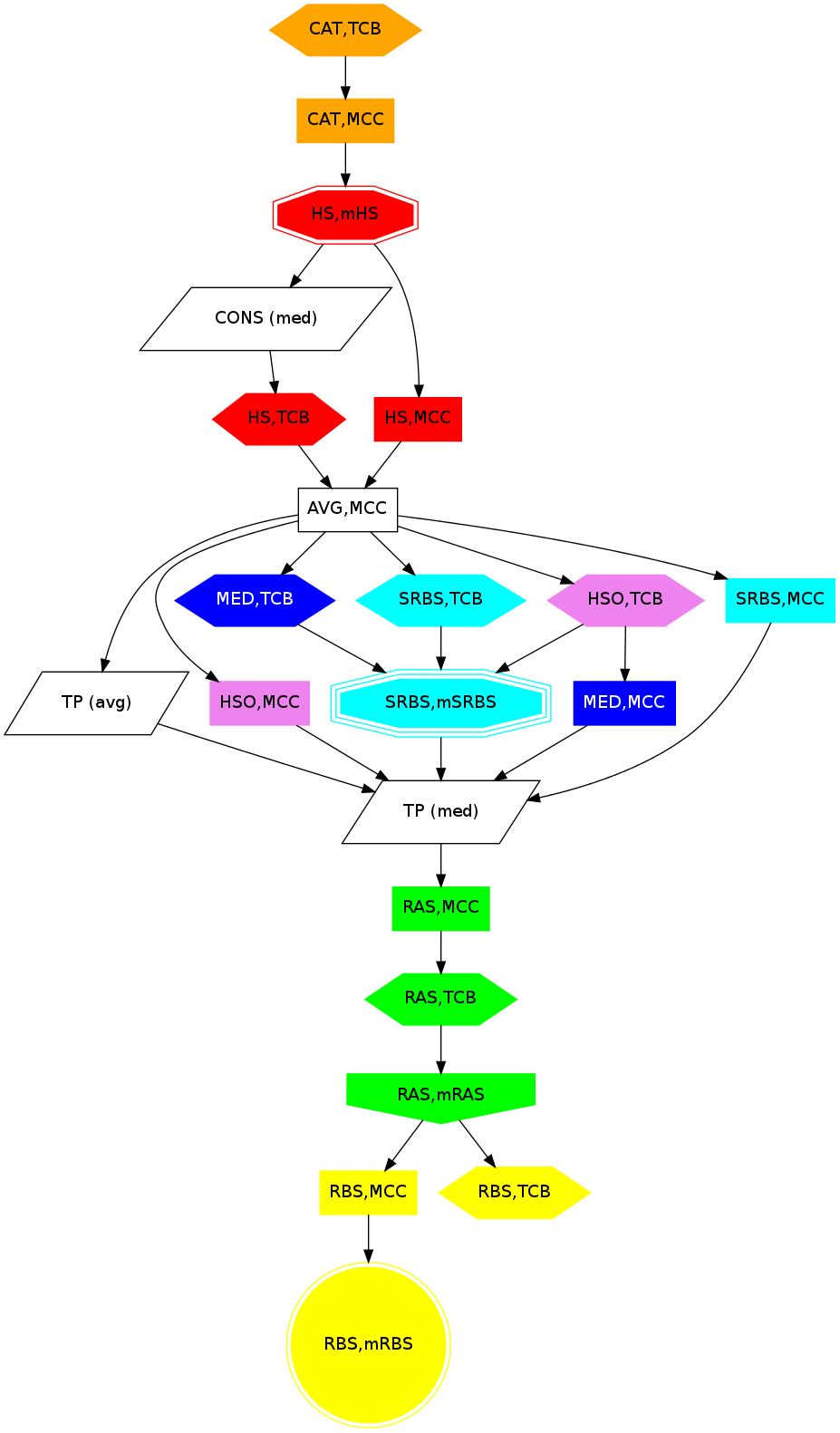

Supplement: Additional file 2 — Posterior summary rank graphs. Method rank graphs for each error measure. [file 1471-2148-13-221-S2.GZ › posteriorSummaryRankGraphs/partial/betterTimesCA.png]

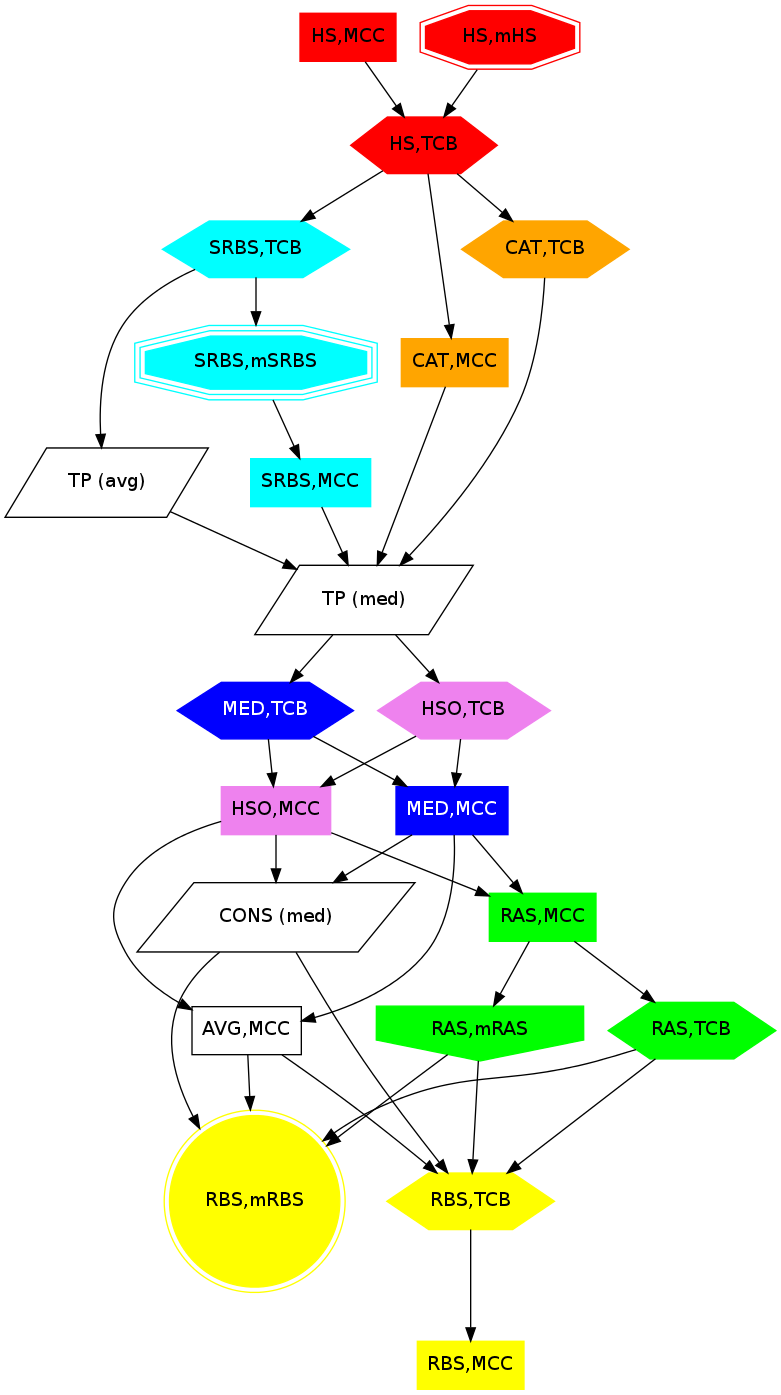

Supplement: Additional file 2 — Posterior summary rank graphs. Method rank graphs for each error measure. [file 1471-2148-13-221-S2.GZ › posteriorSummaryRankGraphs/partial/betterTimesDivergenceV.png]

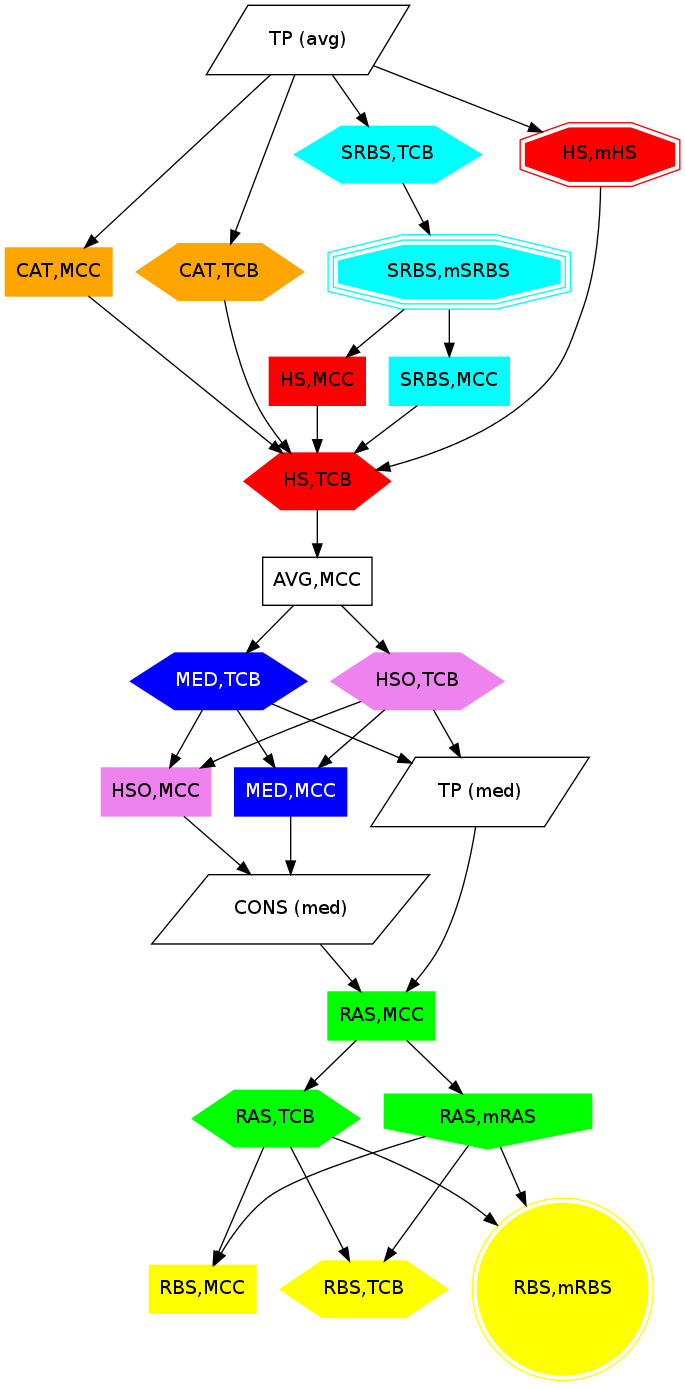

Supplement: Additional file 2 — Posterior summary rank graphs. Method rank graphs for each error measure. [file 1471-2148-13-221-S2.GZ › posteriorSummaryRankGraphs/partial/betterTimesDivergence.png]

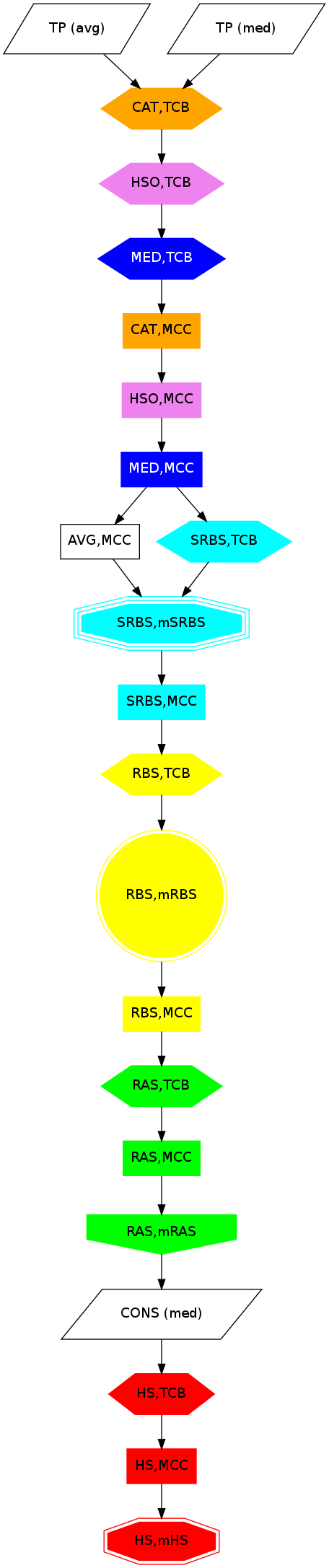

Supplement: Additional file 2 — Posterior summary rank graphs. Method rank graphs for each error measure. [file 1471-2148-13-221-S2.GZ › posteriorSummaryRankGraphs/partial/betterCladeMissesV.png]

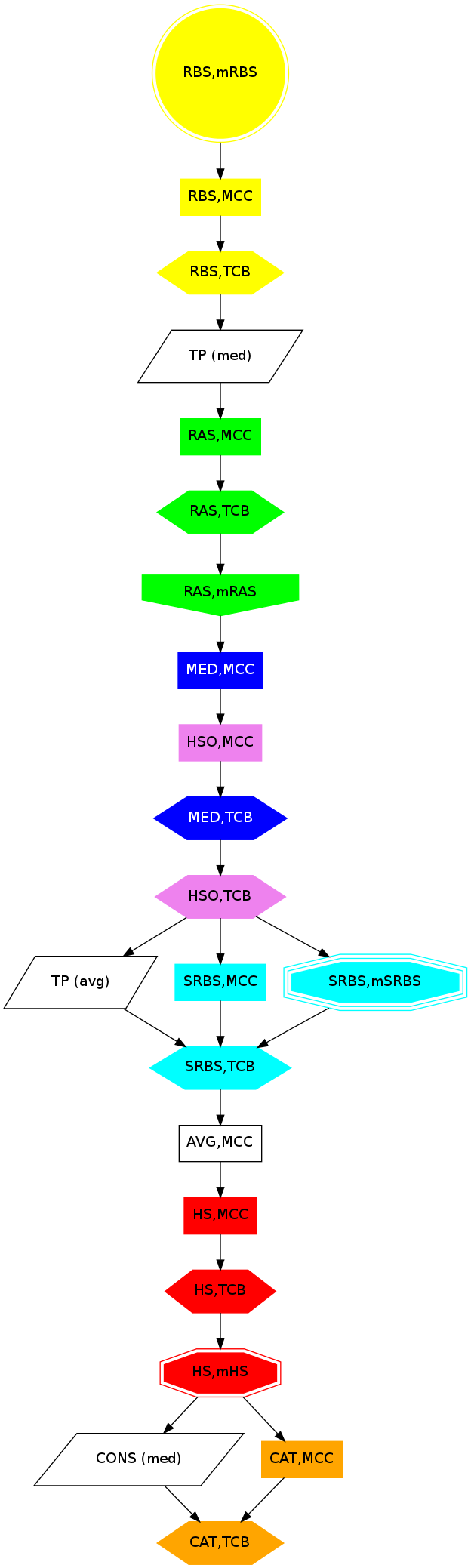

Supplement: Additional file 2 — Posterior summary rank graphs. Method rank graphs for each error measure. [file 1471-2148-13-221-S2.GZ › posteriorSummaryRankGraphs/partial/betterLLmodel.png]

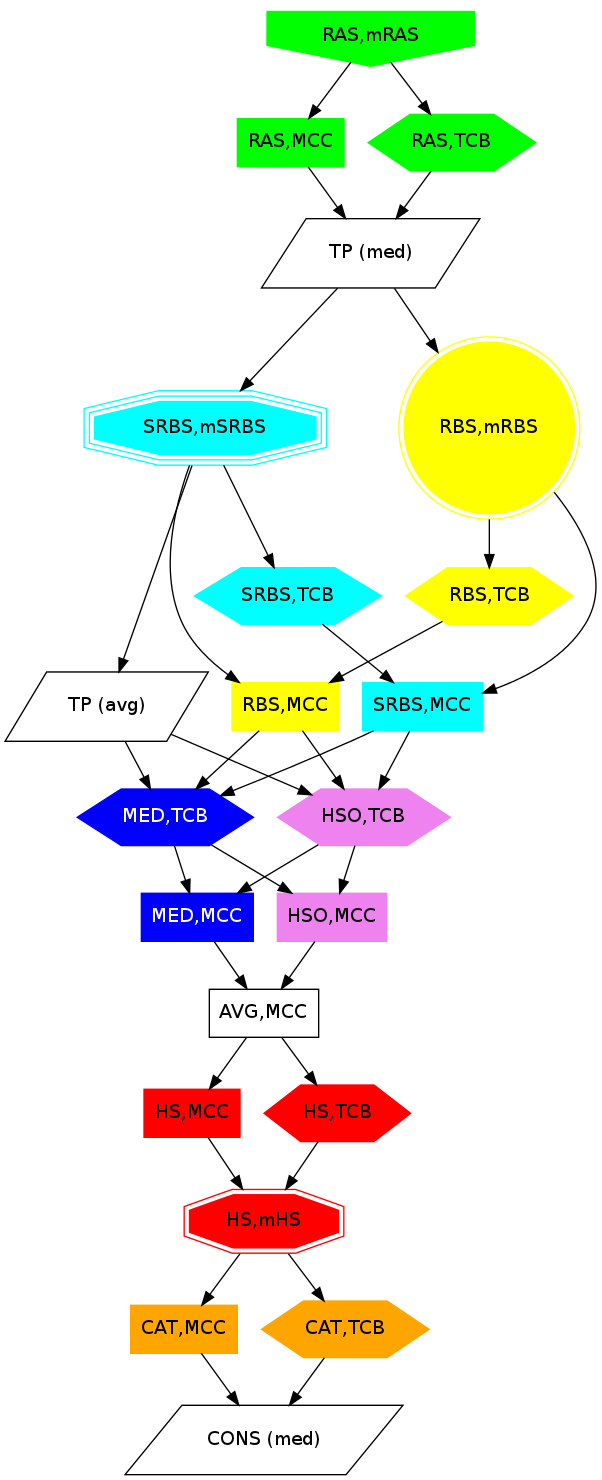

Supplement: Additional file 2 — Posterior summary rank graphs. Method rank graphs for each error measure. [file 1471-2148-13-221-S2.GZ › posteriorSummaryRankGraphs/partial/closer2truthrasV.png]

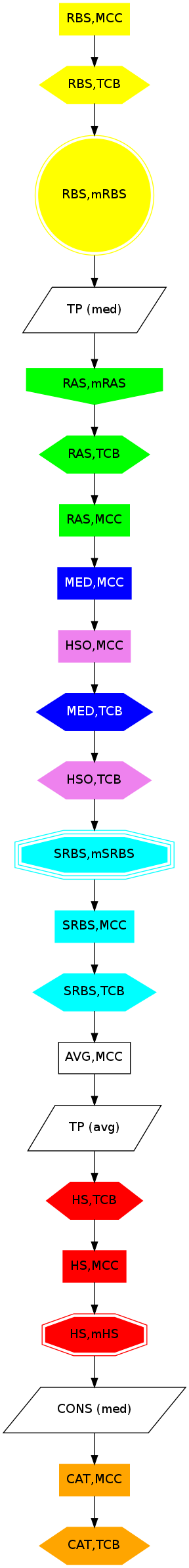

Supplement: Additional file 2 — Posterior summary rank graphs. Method rank graphs for each error measure. [file 1471-2148-13-221-S2.GZ › posteriorSummaryRankGraphs/partial/betterLLcoalV.png]

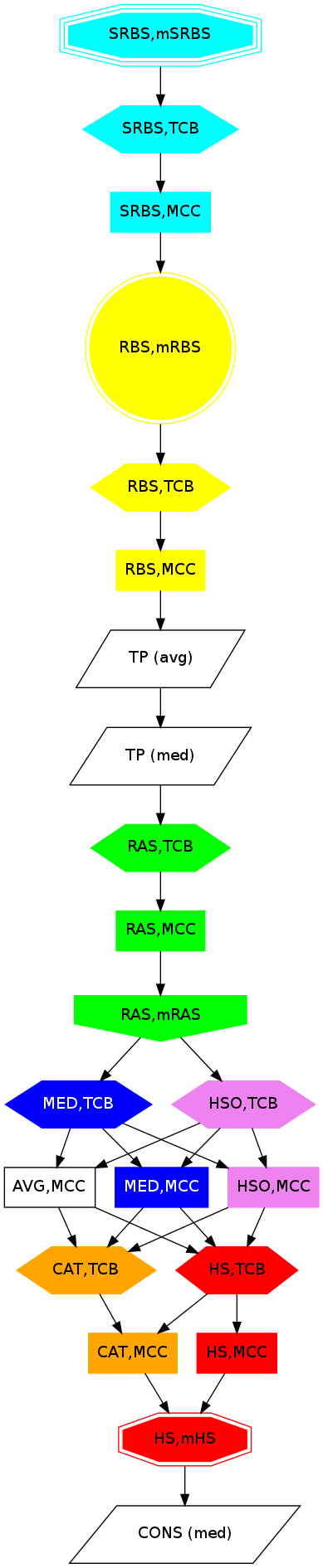

Supplement: Additional file 2 — Posterior summary rank graphs. Method rank graphs for each error measure. [file 1471-2148-13-221-S2.GZ › posteriorSummaryRankGraphs/partial/closer2truthbs2V.png]

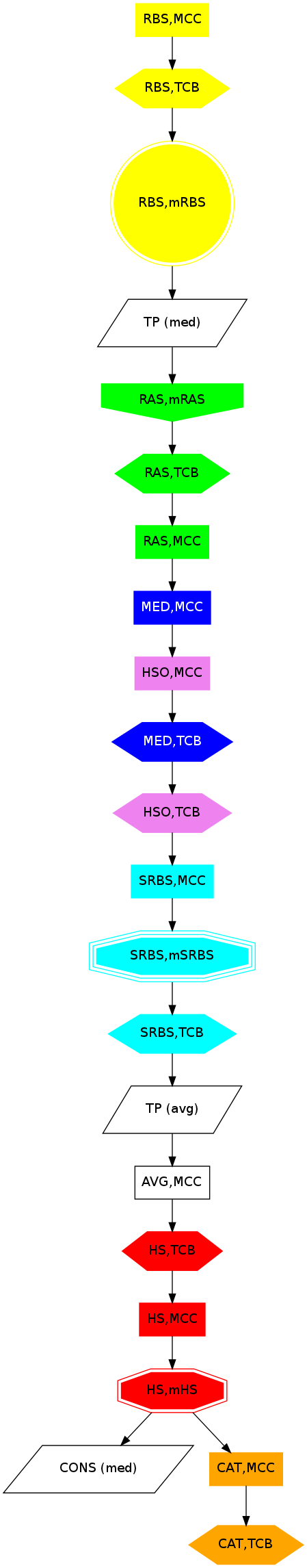

Supplement: Additional file 2 — Posterior summary rank graphs. Method rank graphs for each error measure. [file 1471-2148-13-221-S2.GZ › posteriorSummaryRankGraphs/partial/betterLLcoal.png]

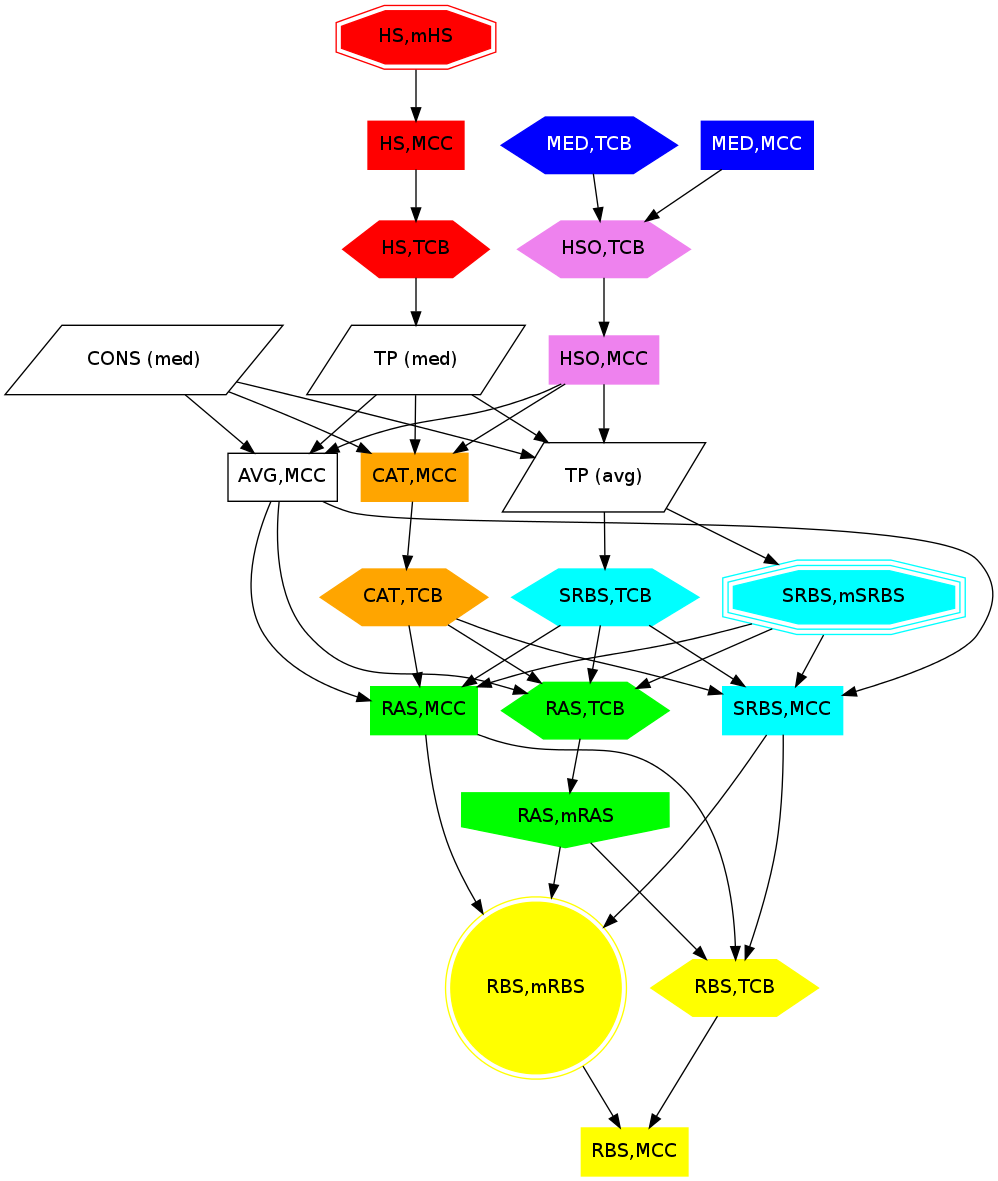

Supplement: Additional file 2 — Posterior summary rank graphs. Method rank graphs for each error measure. [file 1471-2148-13-221-S2.GZ › posteriorSummaryRankGraphs/partial/betterTimesRootV.png]

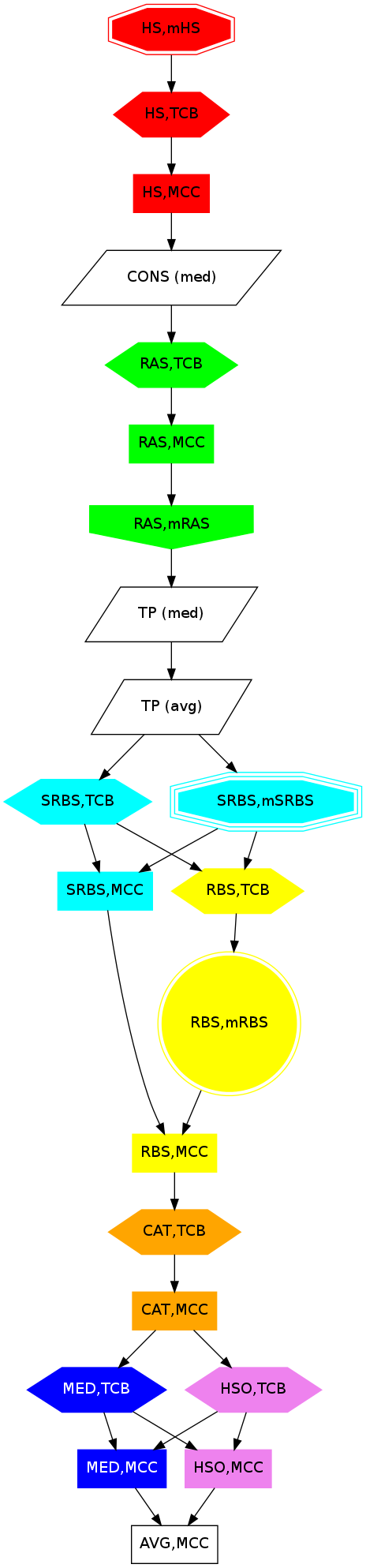

Supplement: Additional file 2 — Posterior summary rank graphs. Method rank graphs for each error measure. [file 1471-2148-13-221-S2.GZ › posteriorSummaryRankGraphs/partial/closer2truthhsV.png]

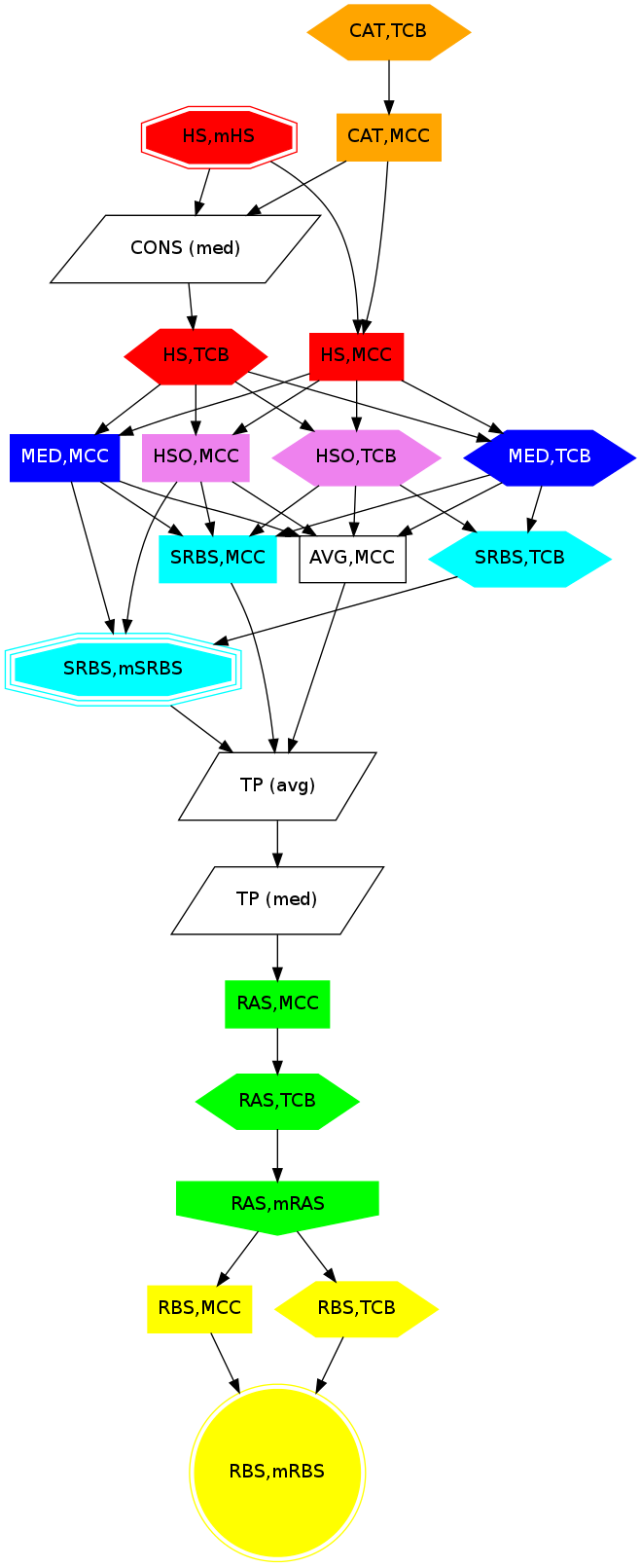

Supplement: Additional file 2 — Posterior summary rank graphs. Method rank graphs for each error measure. [file 1471-2148-13-221-S2.GZ › posteriorSummaryRankGraphs/partial/betterTimesCAV.png]

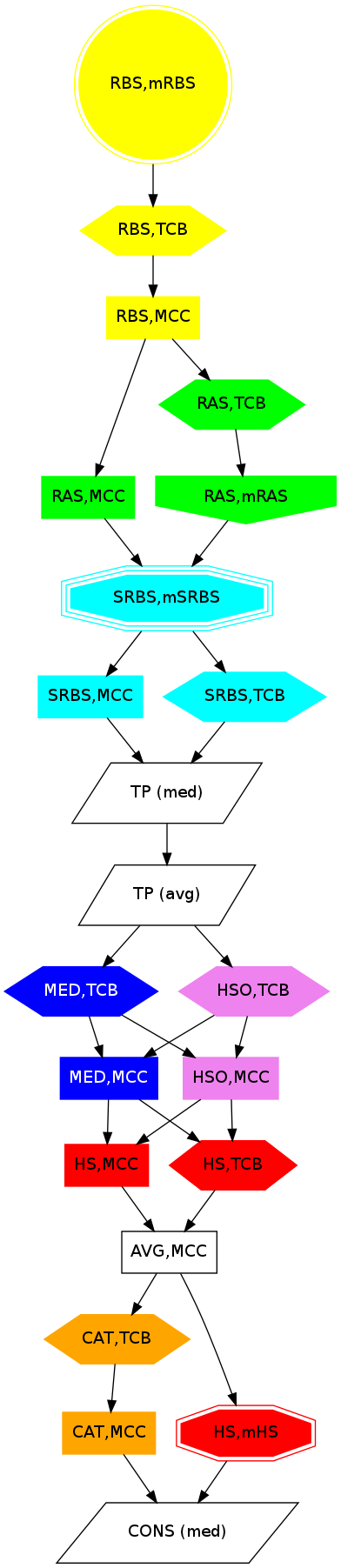

Supplement: Additional file 2 — Posterior summary rank graphs. Method rank graphs for each error measure. [file 1471-2148-13-221-S2.GZ › posteriorSummaryRankGraphs/partial/closer2truthbs1.png]

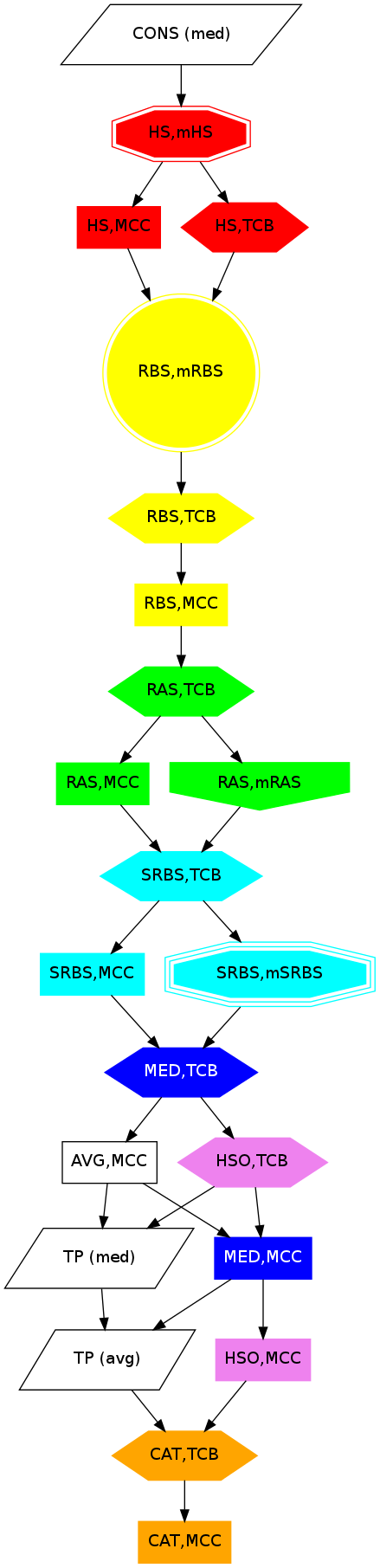

Supplement: Additional file 2 — Posterior summary rank graphs. Method rank graphs for each error measure. [file 1471-2148-13-221-S2.GZ › posteriorSummaryRankGraphs/partial/betterCladeCalls.png]

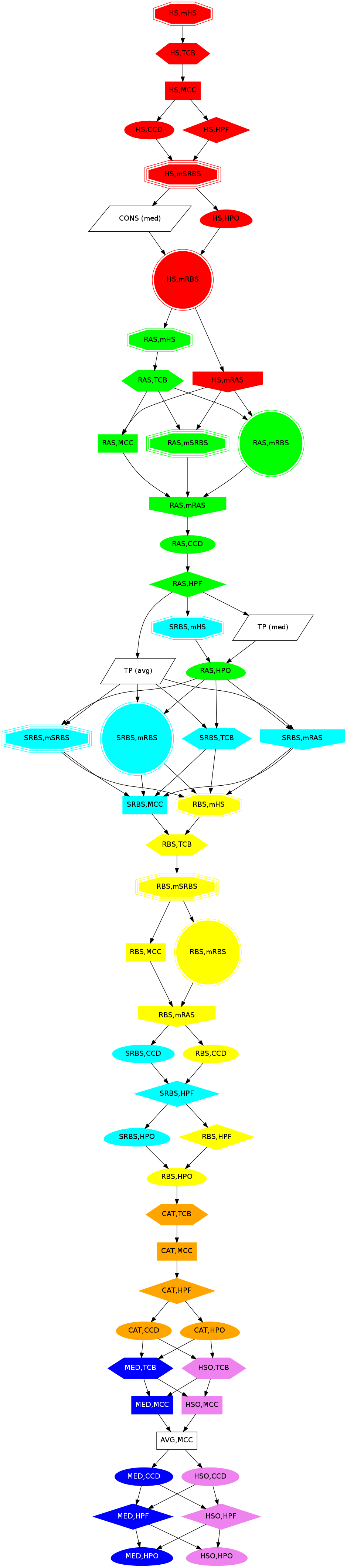

Supplement: Additional file 2 — Posterior summary rank graphs. Method rank graphs for each error measure. [file 1471-2148-13-221-S2.GZ › posteriorSummaryRankGraphs/full/closer2truthhs.png]

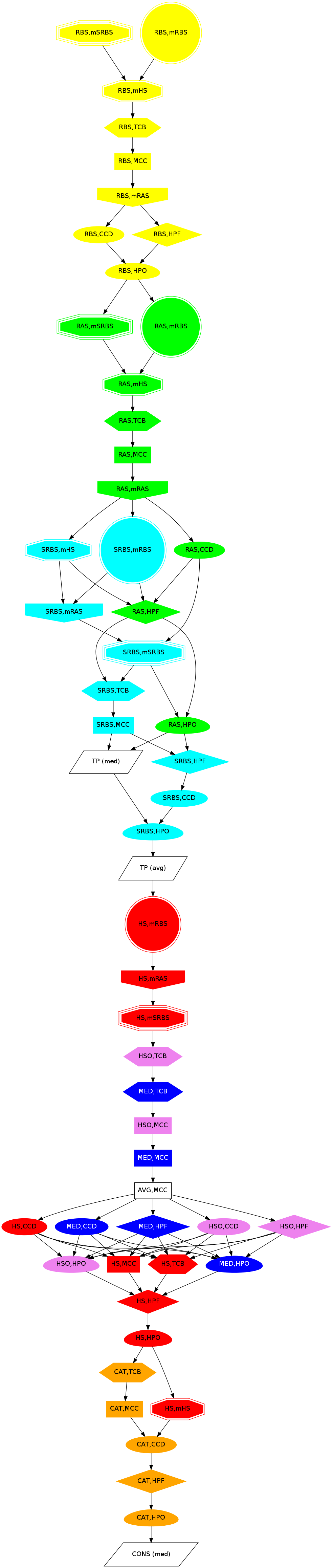

Supplement: Additional file 2 — Posterior summary rank graphs. Method rank graphs for each error measure. [file 1471-2148-13-221-S2.GZ › posteriorSummaryRankGraphs/full/closer2truthbs1V.png]

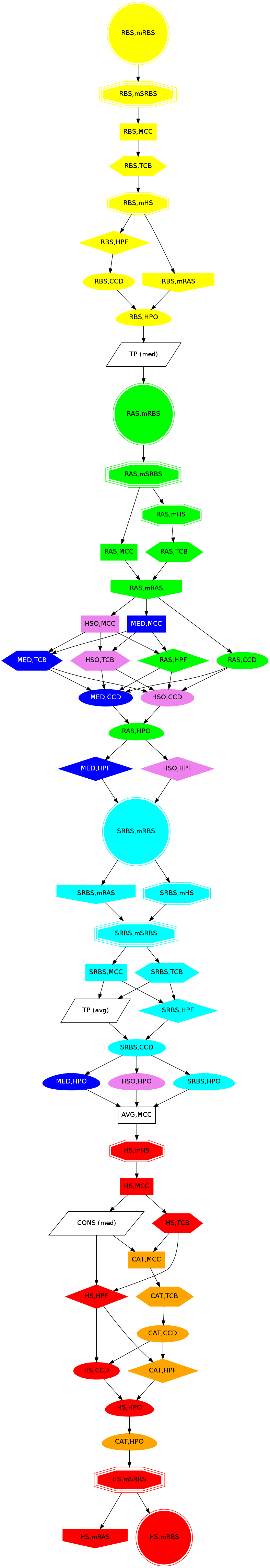

Supplement: Additional file 2 — Posterior summary rank graphs. Method rank graphs for each error measure. [file 1471-2148-13-221-S2.GZ › posteriorSummaryRankGraphs/full/betterLLmodelV.png]

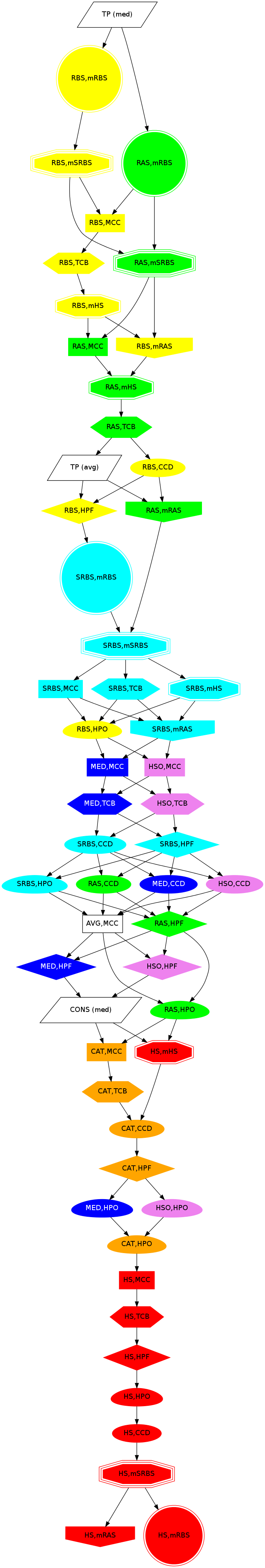

Supplement: Additional file 2 — Posterior summary rank graphs. Method rank graphs for each error measure. [file 1471-2148-13-221-S2.GZ › posteriorSummaryRankGraphs/full/betterLLtreeV.png]

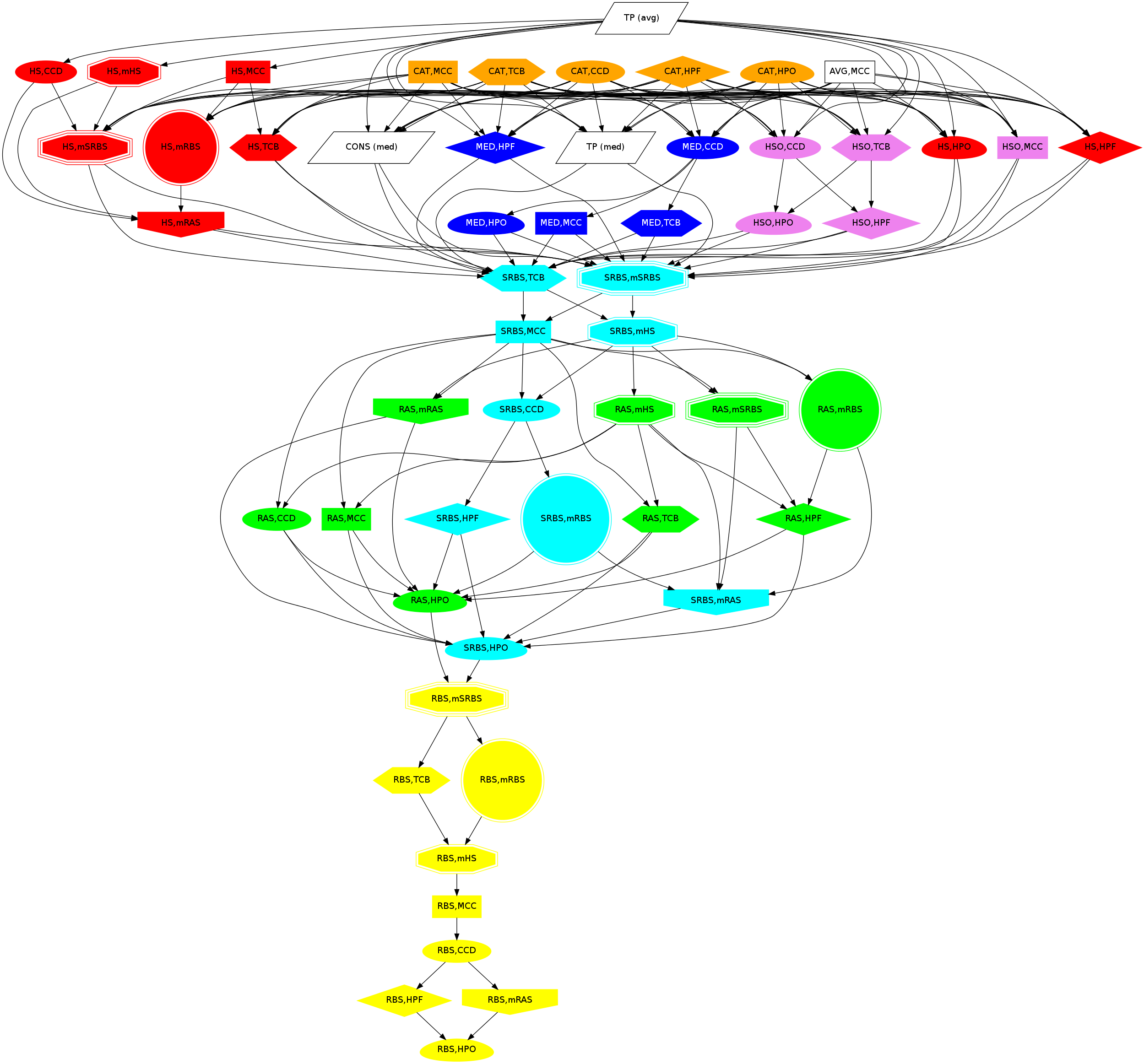

Supplement: Additional file 2 — Posterior summary rank graphs. Method rank graphs for each error measure. [file 1471-2148-13-221-S2.GZ › posteriorSummaryRankGraphs/full/betterTimesRoot.png]

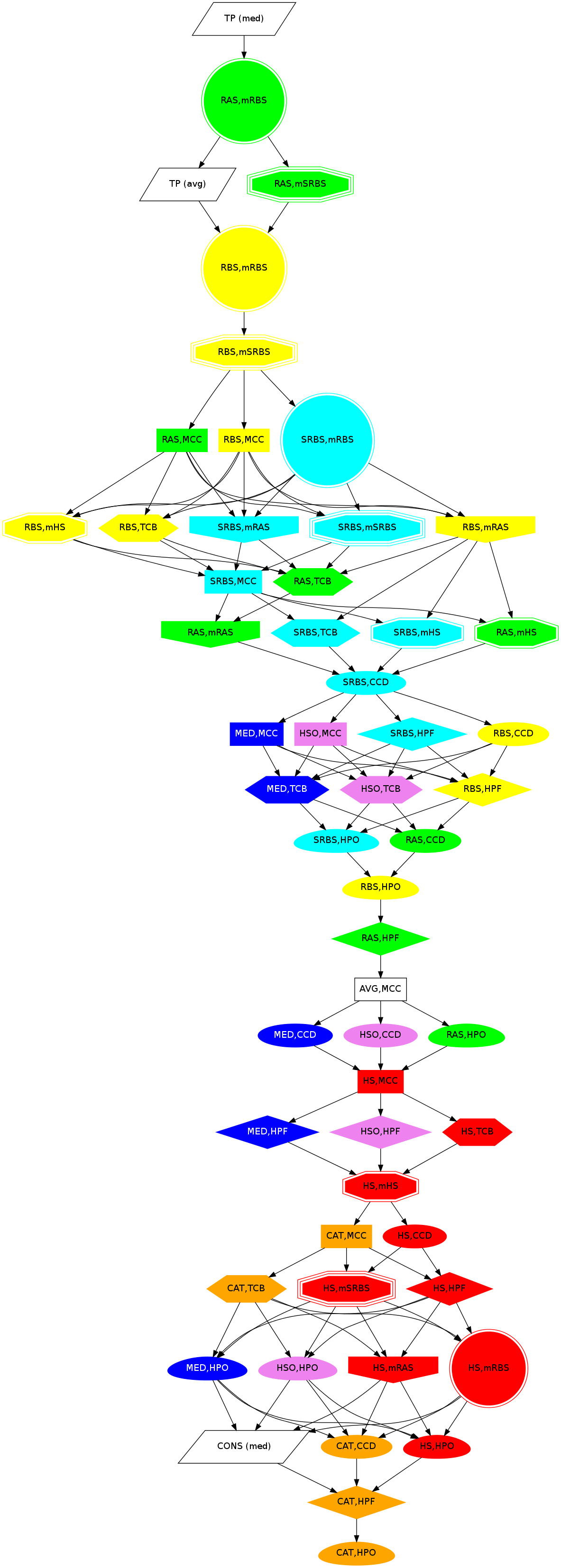

Supplement: Additional file 2 — Posterior summary rank graphs. Method rank graphs for each error measure. [file 1471-2148-13-221-S2.GZ › posteriorSummaryRankGraphs/full/betterLLtree.png]

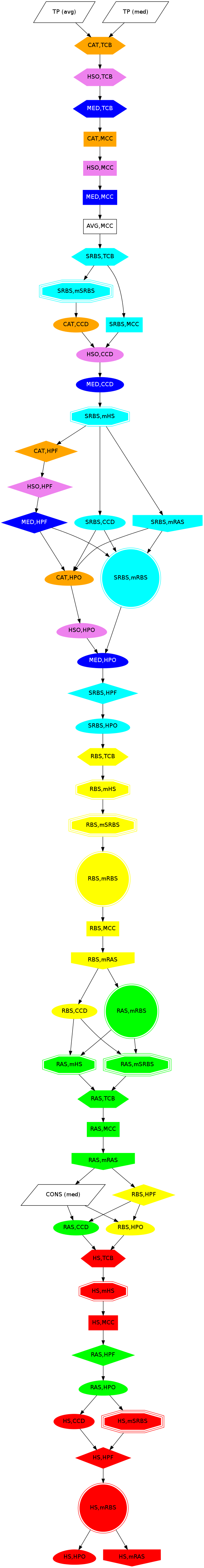

Supplement: Additional file 2 — Posterior summary rank graphs. Method rank graphs for each error measure. [file 1471-2148-13-221-S2.GZ › posteriorSummaryRankGraphs/full/betterCladeMisses.png]

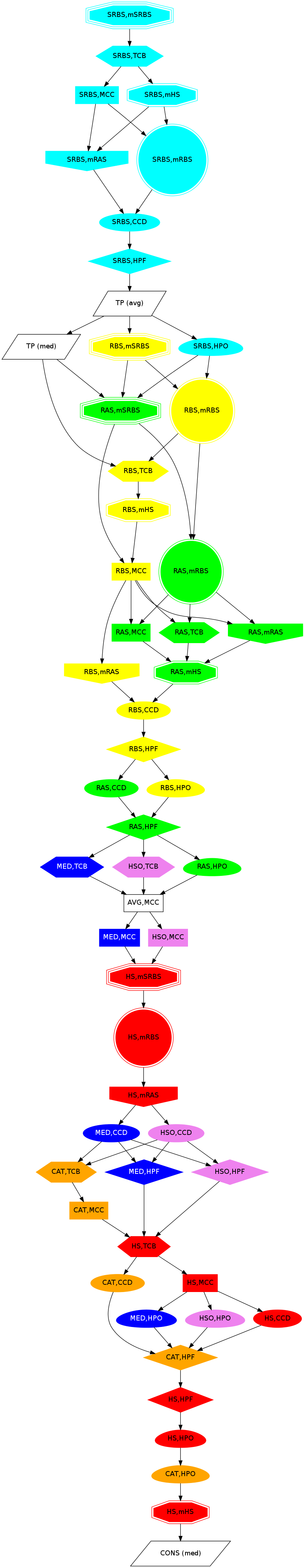

Supplement: Additional file 2 — Posterior summary rank graphs. Method rank graphs for each error measure. [file 1471-2148-13-221-S2.GZ › posteriorSummaryRankGraphs/full/closer2truthbs2.png]

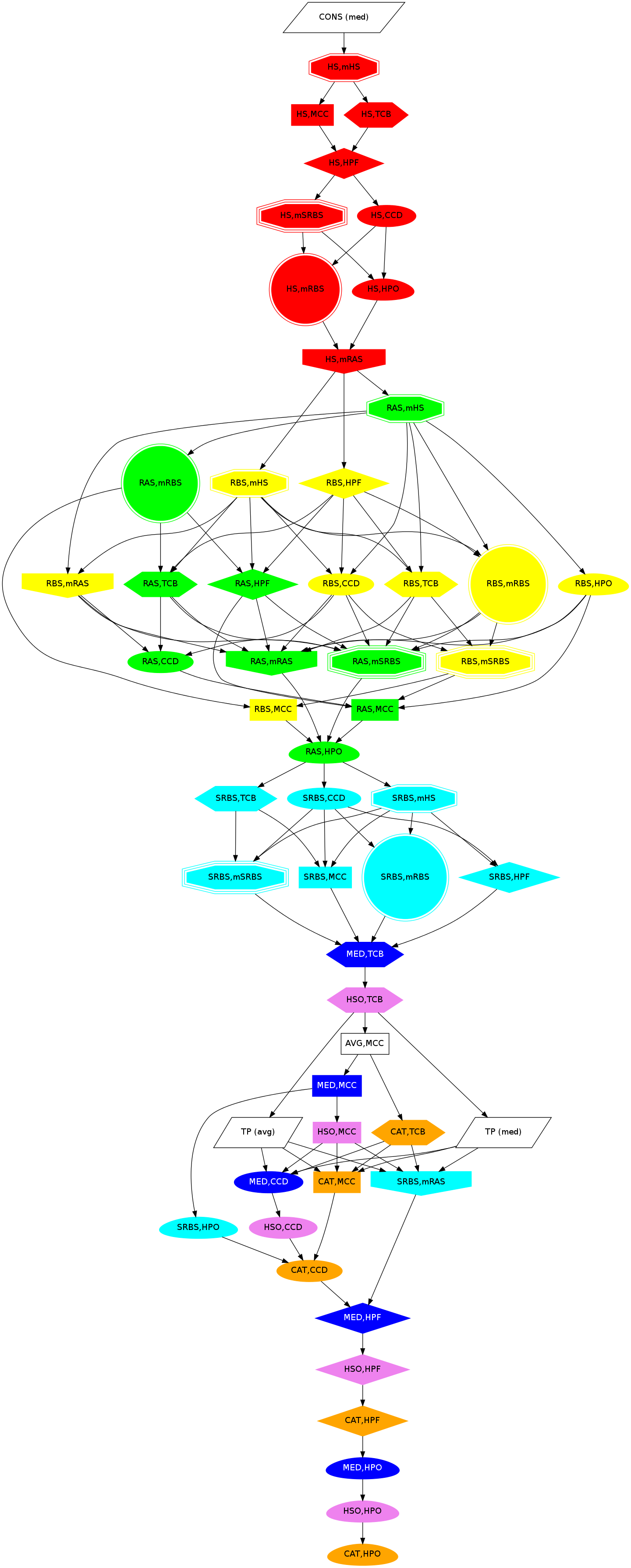

Supplement: Additional file 2 — Posterior summary rank graphs. Method rank graphs for each error measure. [file 1471-2148-13-221-S2.GZ › posteriorSummaryRankGraphs/full/betterCladeCallsV.png]

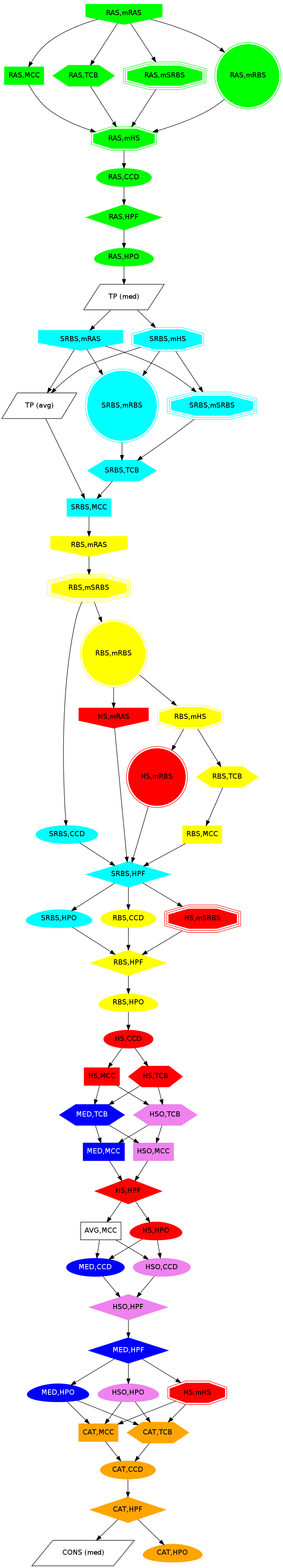

Supplement: Additional file 2 — Posterior summary rank graphs. Method rank graphs for each error measure. [file 1471-2148-13-221-S2.GZ › posteriorSummaryRankGraphs/full/closer2truthras.png]

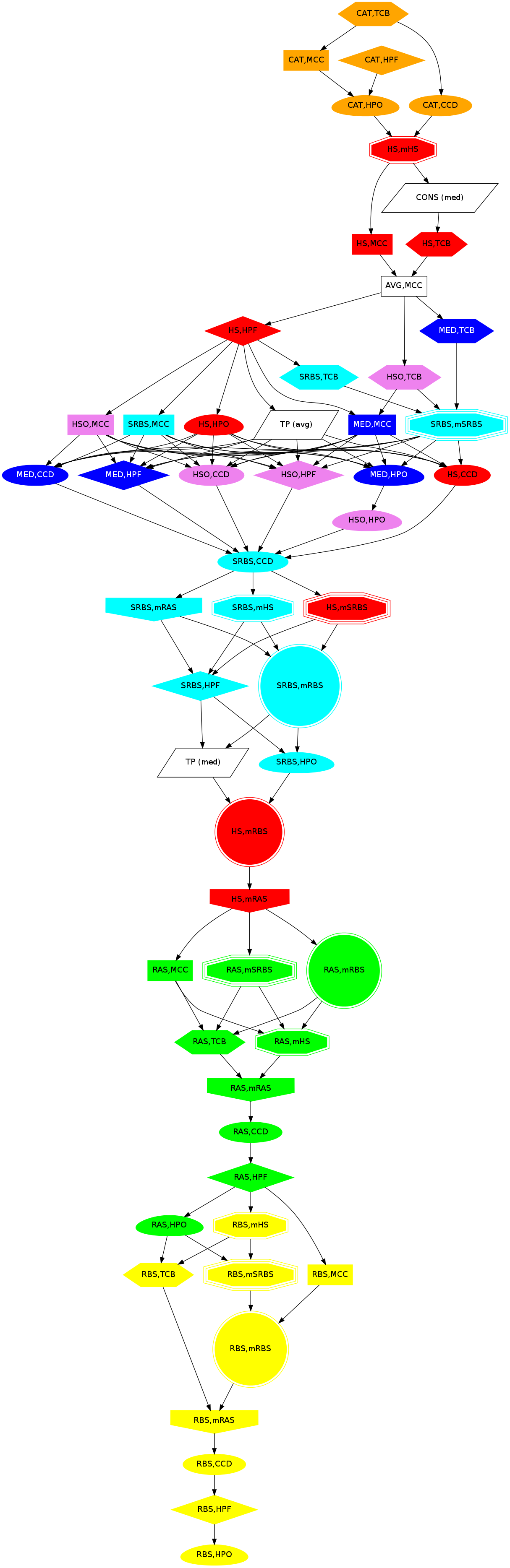

Supplement: Additional file 2 — Posterior summary rank graphs. Method rank graphs for each error measure. [file 1471-2148-13-221-S2.GZ › posteriorSummaryRankGraphs/full/betterTimesCA.png]

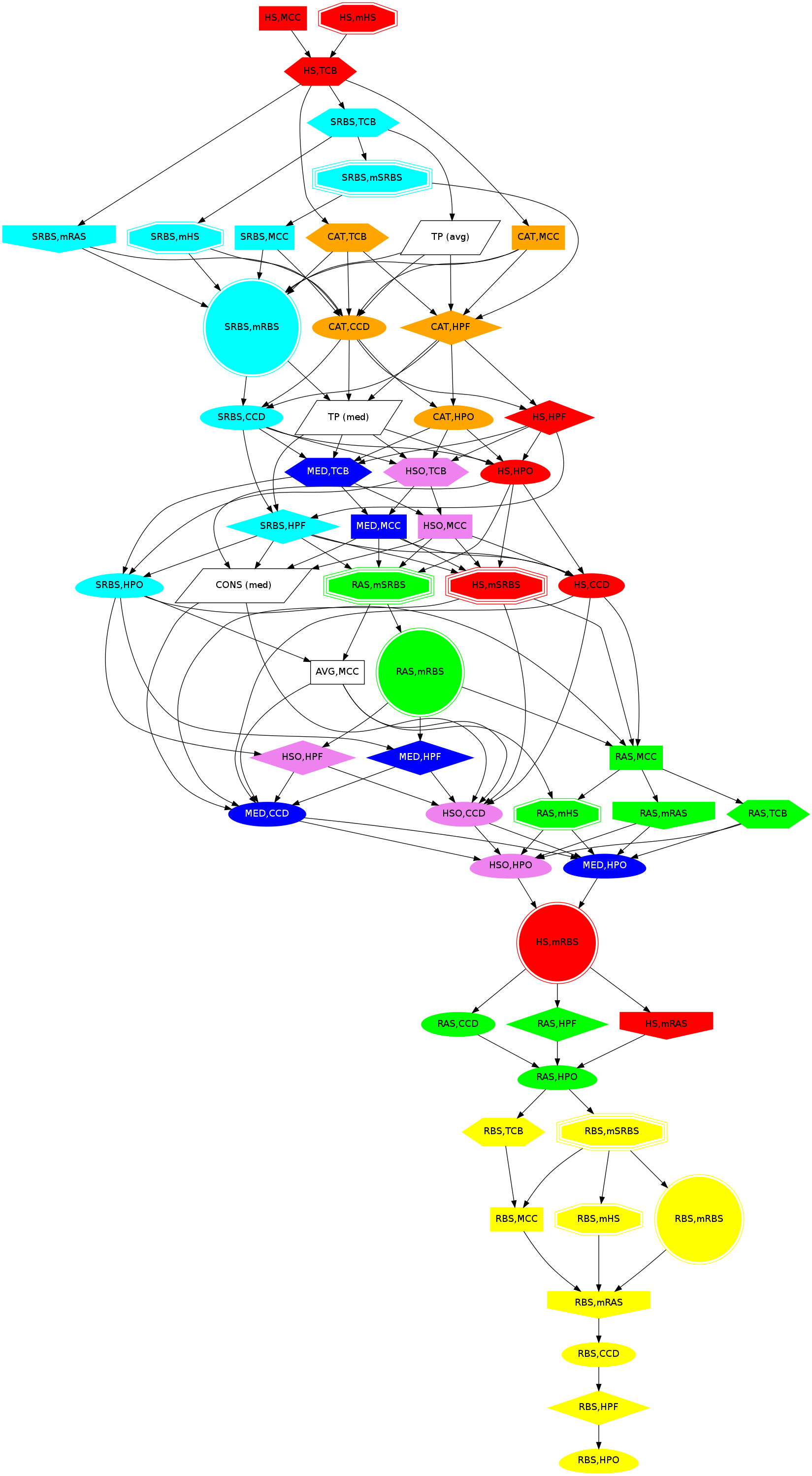

Supplement: Additional file 2 — Posterior summary rank graphs. Method rank graphs for each error measure. [file 1471-2148-13-221-S2.GZ › posteriorSummaryRankGraphs/full/betterTimesDivergenceV.png]

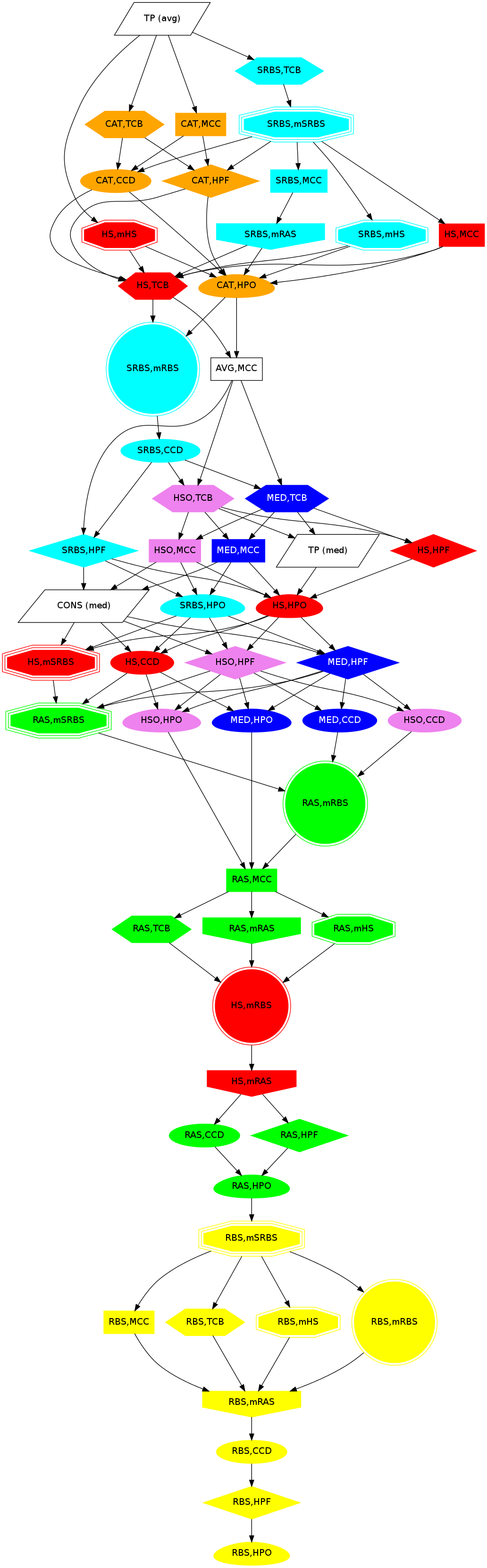

Supplement: Additional file 2 — Posterior summary rank graphs. Method rank graphs for each error measure. [file 1471-2148-13-221-S2.GZ › posteriorSummaryRankGraphs/full/betterTimesDivergence.png]

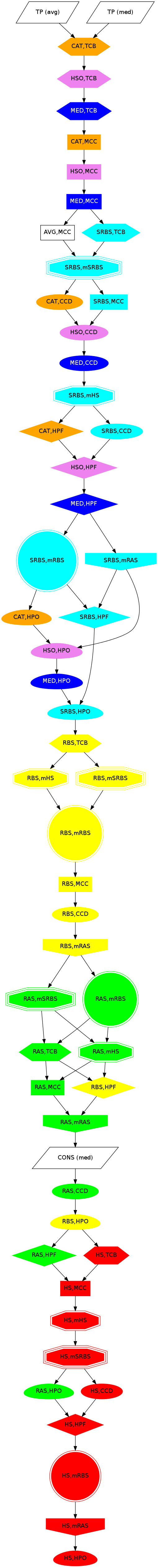

Supplement: Additional file 2 — Posterior summary rank graphs. Method rank graphs for each error measure. [file 1471-2148-13-221-S2.GZ › posteriorSummaryRankGraphs/full/betterCladeMissesV.png]

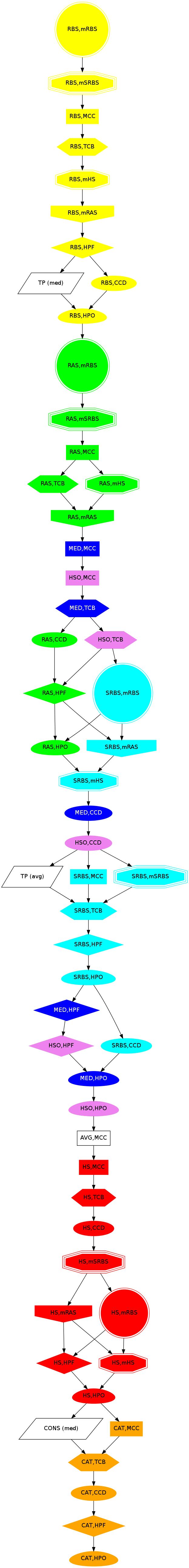

Supplement: Additional file 2 — Posterior summary rank graphs. Method rank graphs for each error measure. [file 1471-2148-13-221-S2.GZ › posteriorSummaryRankGraphs/full/betterLLmodel.png]

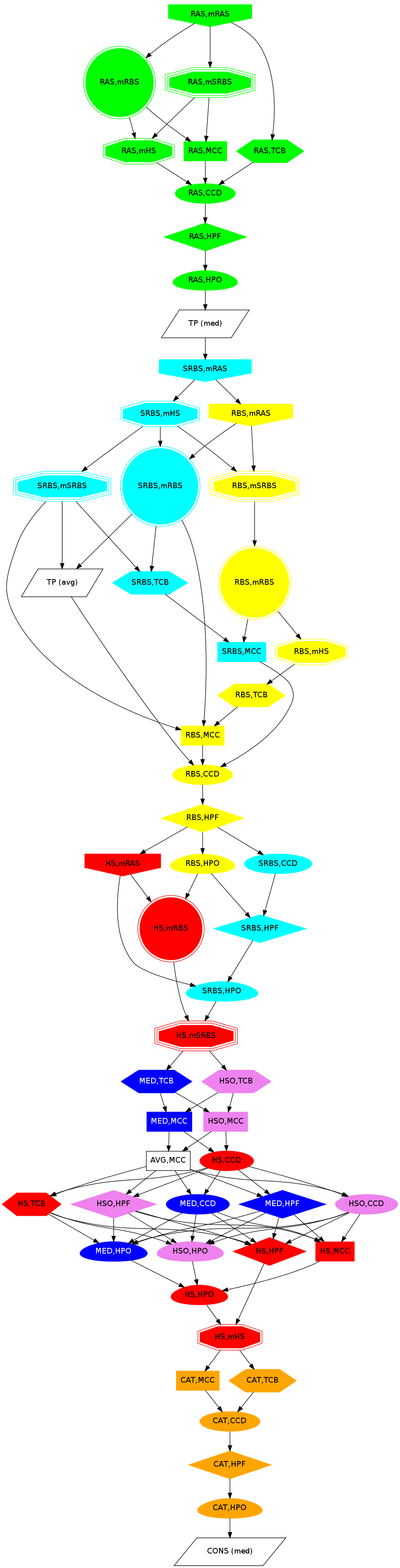

Supplement: Additional file 2 — Posterior summary rank graphs. Method rank graphs for each error measure. [file 1471-2148-13-221-S2.GZ › posteriorSummaryRankGraphs/full/closer2truthrasV.png]

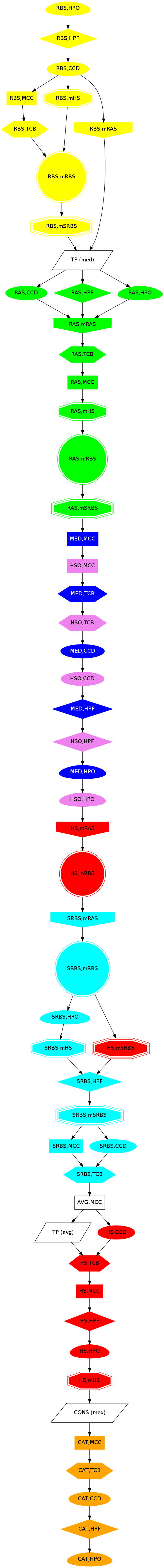

Supplement: Additional file 2 — Posterior summary rank graphs. Method rank graphs for each error measure. [file 1471-2148-13-221-S2.GZ › posteriorSummaryRankGraphs/full/betterLLcoalV.png]

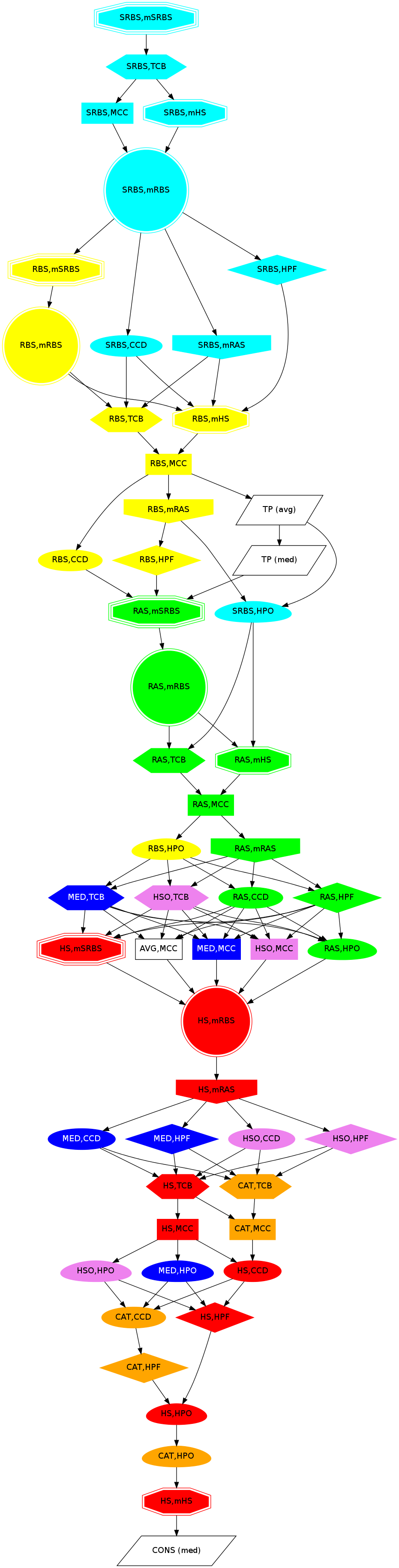

Supplement: Additional file 2 — Posterior summary rank graphs. Method rank graphs for each error measure. [file 1471-2148-13-221-S2.GZ › posteriorSummaryRankGraphs/full/closer2truthbs2V.png]

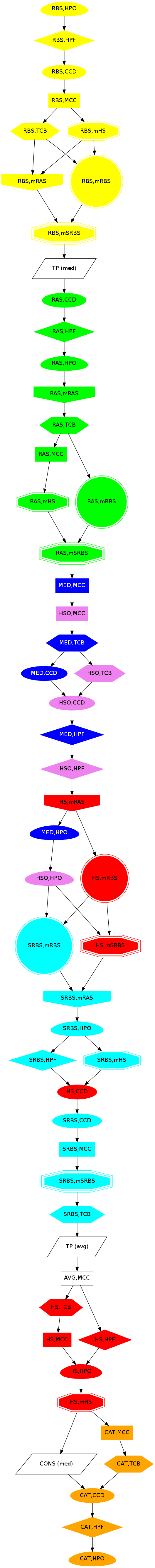

Supplement: Additional file 2 — Posterior summary rank graphs. Method rank graphs for each error measure. [file 1471-2148-13-221-S2.GZ › posteriorSummaryRankGraphs/full/betterLLcoal.png]

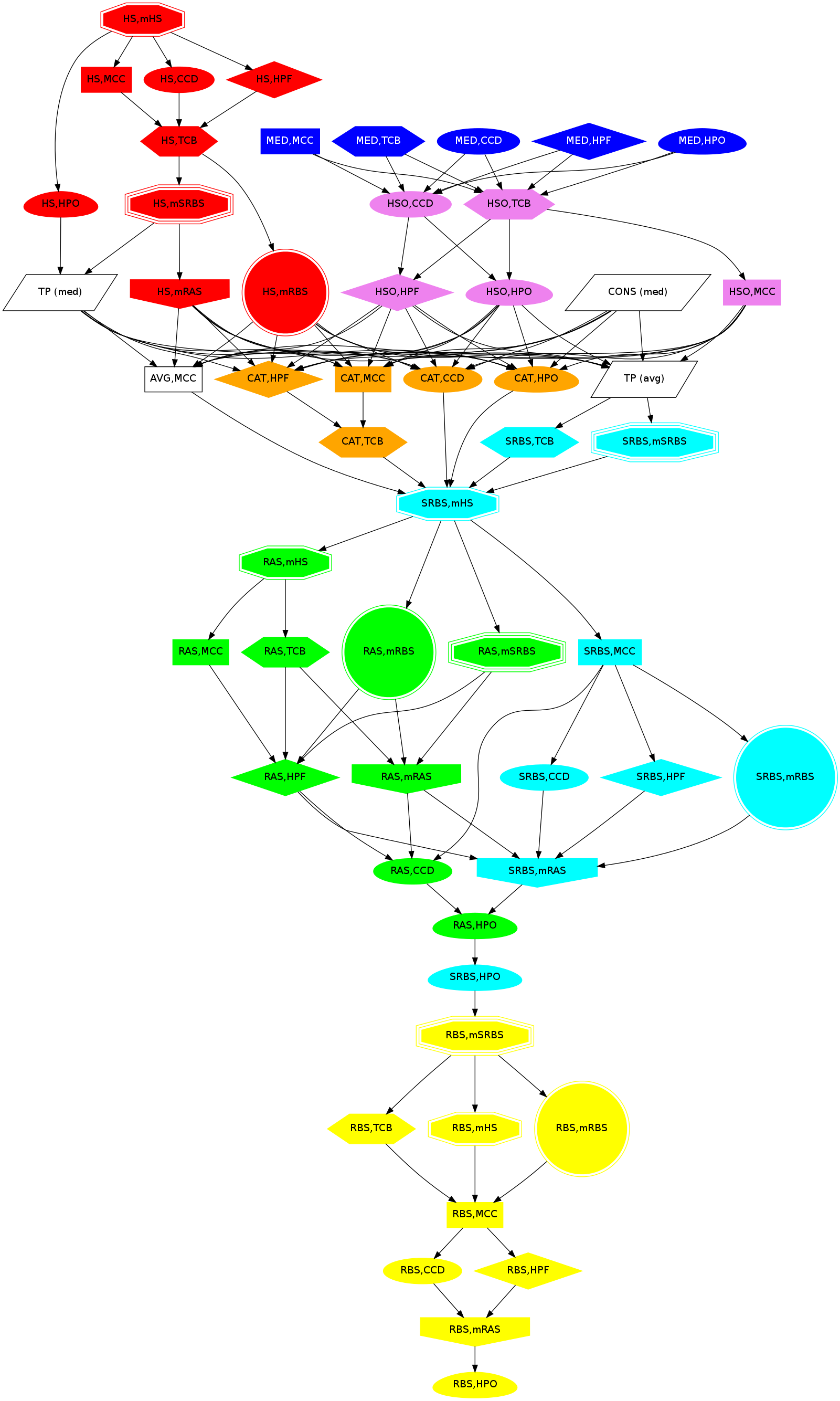

Supplement: Additional file 2 — Posterior summary rank graphs. Method rank graphs for each error measure. [file 1471-2148-13-221-S2.GZ › posteriorSummaryRankGraphs/full/betterTimesRootV.png]

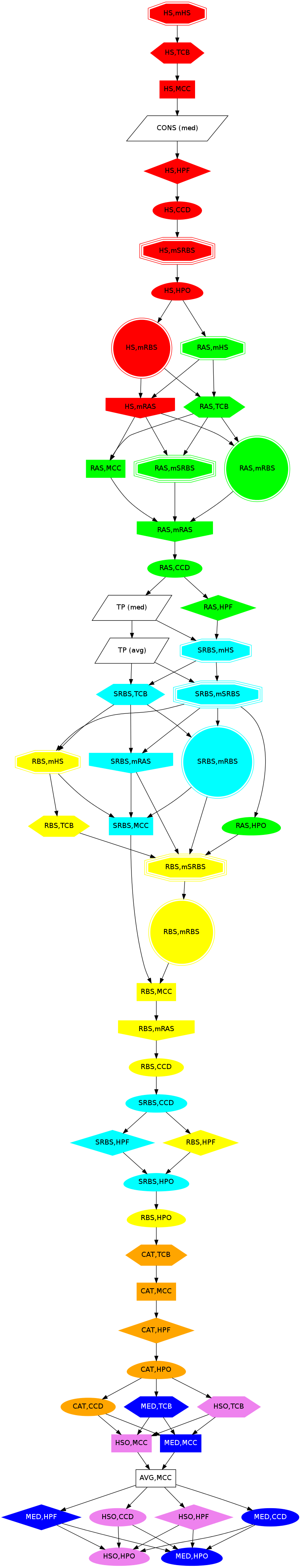

Supplement: Additional file 2 — Posterior summary rank graphs. Method rank graphs for each error measure. [file 1471-2148-13-221-S2.GZ › posteriorSummaryRankGraphs/full/closer2truthhsV.png]

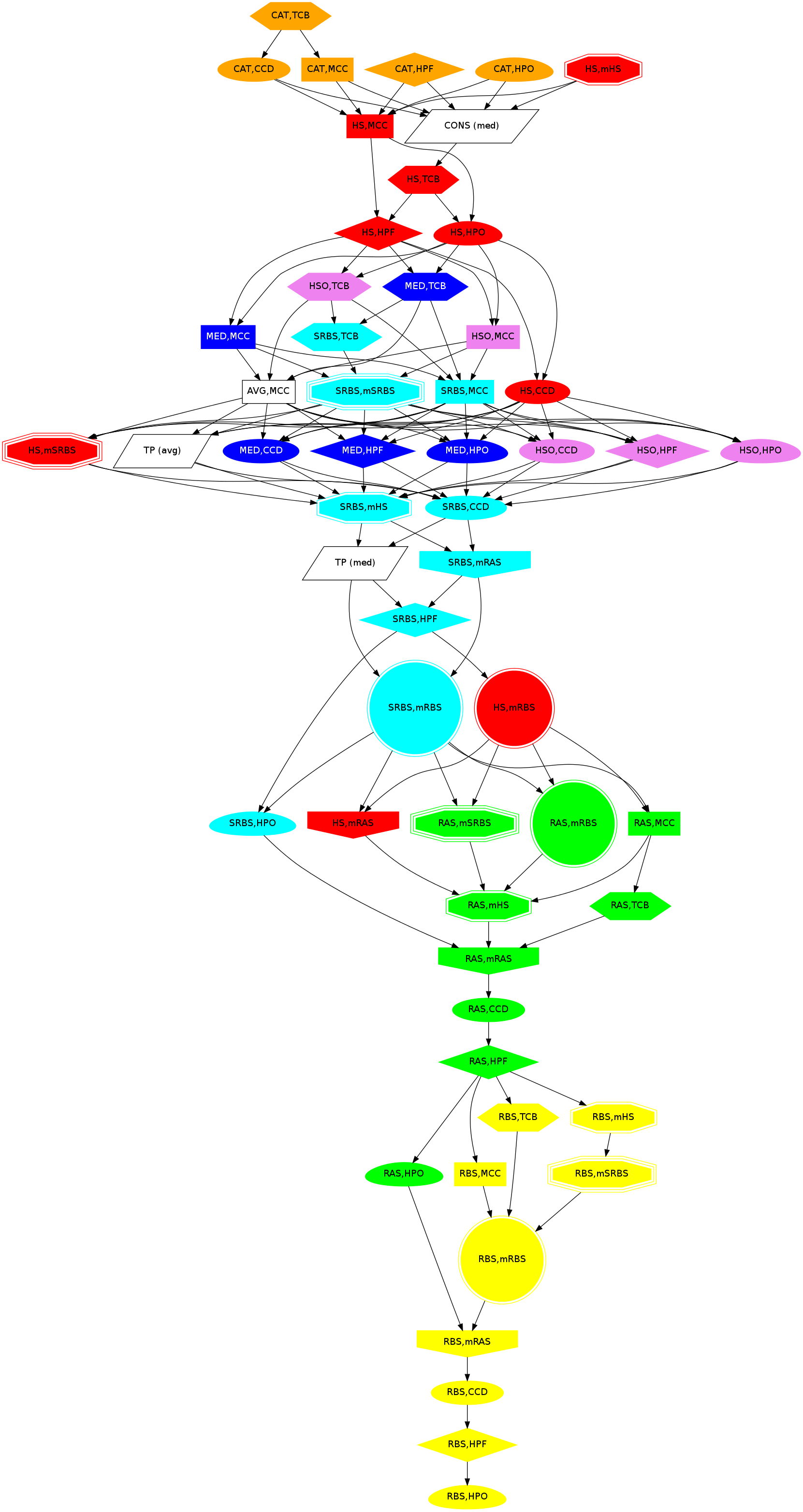

Supplement: Additional file 2 — Posterior summary rank graphs. Method rank graphs for each error measure. [file 1471-2148-13-221-S2.GZ › posteriorSummaryRankGraphs/full/betterTimesCAV.png]

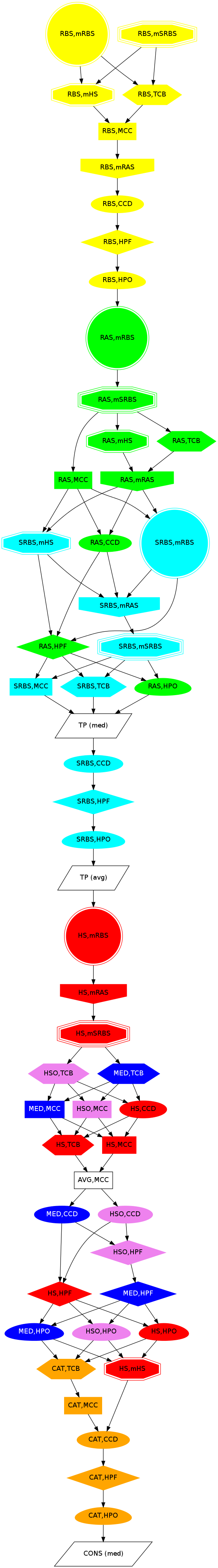

Supplement: Additional file 2 — Posterior summary rank graphs. Method rank graphs for each error measure. [file 1471-2148-13-221-S2.GZ › posteriorSummaryRankGraphs/full/closer2truthbs1.png]

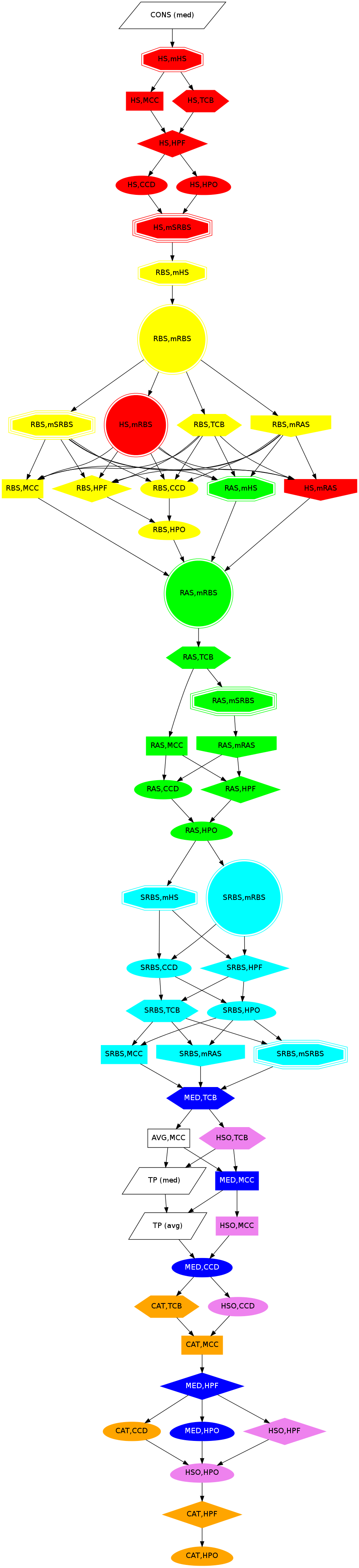

Supplement: Additional file 2 — Posterior summary rank graphs. Method rank graphs for each error measure. [file 1471-2148-13-221-S2.GZ › posteriorSummaryRankGraphs/full/betterCladeCalls.png]
